# Computing double-pushout graph transformation rules and atom-to-atom maps from KEGG RCLASS data

**DOI:** 10.1186/s13015-025-00294-6

**Published:** 2026-01-29

**Authors:** Nora Beier, Thomas Gatter, Jakob L. Andersen, Peter F. Stadler

**Affiliations:** 1 Bioinformatics Group, Department of Computer Science, and Interdisciplinary Center for Bioinformatics, Universität Leipzig, Härtelstraße 16–18, D-04107 Leipzig, Germany; 2 Max Planck Institute for Mathematics in the Sciences, Inselstraße 22, D-04103 Leipzig, Germany; 3 Department of Theoretical Chemistry, University of Vienna, Währinger Straße 17, Vienna, A-1090 Austria; 4 Faculdad de Ciencias, Universidad Nacional de Colombia, Sede Bogotá, Ciudad Universitaria, Bogotá, D.C. COL-111321 Colombia; 5 Santa Fe Institute, 1399 Hyde Park Rd., Santa Fe, NM87501 USA; 6 Department of Mathematics and Computer Science, University of Southern Denmark, Campusvej 55, 5230 Odense M, Denmark; 7 Center for non-coding RNA in Technology and Health, University of Copenhagen, Frederiksberg, Denmark

**Keywords:** Atom-to-Atom mapping, RCLASS, KEGG, Double push out rules, Graph reconstruction, Graph transformation, Metabolic reactions, Reaction rules

## Abstract

**Background:**

Atom-to-atom maps play an important role in many applications. However, they are often difficult to obtain. The KEGG reaction database does not provide atom-to-atom maps for its reactions and instead offers a description of local changes for pairs of reactant and product molecules in terms of so-called RCLASSes. Developed for classification purposes, RCLASS data are difficult to use for purposes such as the construction of atom-to-atom maps or reaction rules. DPO graph transformation rules, on the other hand, work as a convenient and efficient representation, particularly for these applications. The RCLASS data can be understood as collections of local graph patterns in the reactants and products of a reaction, together with partial correspondences of atoms. The problem of converting RCLASS data into DPO rules, therefore, is a special case of the graph reconstruction problem, which consists of inferring a graph from a collection of subgraphs.

**Results:**

We developed laveau, a tool that computes explicit DPO rules from KEGG reactions and RCLASS data. The algorithm proceeds stepwise, starting with a translation of individual RDM codes, specifically developed by the KEGG database, into equivalent *RDM pattern graphs*. Multiple *RDM pattern graphs* for the same RCLASS are then combined based on their embeddings into the reactant and product molecules, observing certain consistency conditions. In the final step, these combined pairwise patterns are merged into a pair of subgraphs of reactants and products, respectively. If RCLASSes connecting all pairs of reactant and product molecules are available, the complete reaction center(s) is/are contained in the union of these subgraphs. The atom-to-atom map inherited from the RDM codes then defines a DPO transformation rule. Application of these rules to the reactants then yields complete atom-to-atom maps (AAMs). Starting from 3195 RCLASSes, laveau generates a total of 1232 DPO rules and 1594 AAMs.

**Conclusions:**

The laveau software makes it possible to extract local atom-to-atom maps from the RCLASSes of the KEGG database, covering a large set of enzyme-catalyzed reactions. The results are made available in the form of DPO rules for use in atom-level models of metabolic networks, filling a crucial gap in the available data.

## Background

An important problem in cheminformatics is the inference of atom-to-atom maps (AAM) [1–3]. For enzyme-catalyzed reactions, this is particularly difficult because the mechanisms are often complex and involve atoms of the enzymes in intermediate stages that are not visible in the overall reaction [4]. As a consequence, they differ significantly from uncatalyzed chemical reactions and thus are outside the training scope of the AAM-prediction tools. In a recent study, we observed that RDTool outperformed modern machine learning approaches but is itself limited to an accuracy below 65% [5]. Expert-curated AAM data for biochemical reactions are, therefore, a valuable resource that at present cannot be replaced by purely computational methods.

The KEGG database [6], as one of the key resources for metabolic network data, provides detailed information on enzyme-catalyzed biochemical reactions. It does not provide explicit atom-to-atom maps, however. Instead, an implicit, pattern-based description, called RCLASS, provides at least partial information on the reaction mechanism. At the lowest level, each RCLASS is specified by a set of so-called RDM codes [7], each of which specifies a correspondence between a pattern in one reactant molecule and a corresponding pattern in one product molecule. In this representation, each atom is described by compositional information on its neighborhood, which is encoded by a label that assigns the atom to one of 68 different neighborhood classes [8]. This type of information is available usually only for a subset of all pairs of molecules involved in a given reaction. On the other hand, multiple reactions that share the same mechanism are listed for most RCLASSes. This representation is very difficult to use for purposes other than the classification of reactions [7, 9, 10]. In particular, it is a non-trivial task to retrieve the partial atom-to-atom map underlying an RCLASS.

Graph grammars, on the other hand, naturally capture a reaction mechanism as transformation rules. In the DPO graph transformation [11], each rule is specified as a span $L\leftarrow K\rightarrow R$ in the category of vertex- and edge-labeled graphs, where *L* and *R* are the patterns in reactant and product that are transformed into each other, and *K* is an unchanged context that is isomorphic to a subgraph in both *L* and *R*. The application of a rule to a graph *G* consists of (1) finding a subgraph isomorphism of the reactant pattern *L* in *G*, and (2) deleting from the copy of *L* in *G* all parts not contained in *K*, resulting in a graph *D*, and then gluing in all parts of *R* that are not already present in *K* into *D*. The result is a graph *H* that can be thought of as a copy of *G* in which the pattern *L* is replaced by the right-hand-side *R* of the rule. For a more formal description of DPO graph transformation, we refer to the Theory section below.

In applications to chemical reactions, all atoms are preserved, and thus transformation is limited to inserting, deleting, and changing chemical bonds, i.e., edges in the graphs. The context graph *K* in particular covers all atoms that appear in both *R* and *L* and thus establishes the atom-to-atom map for patterns involved in the reaction. *K* may also contain a set of bonds that remain unchanged between *R* and *L*. Since all atoms are preserved, the application of the rule $L\leftarrow K\rightarrow R$ determines an atom-to-atom map between the reactant graph *G* and the product graph *H*, as well as the intermediate graph *D*. DPO graph transformation is particularly well-suited to model chemistry because it (1) ensures logical reversibility of all rules and reactions and (2) does not cause side-effects [11, 12]. DPO graph transformation is implemented in the software package MØD [13, 14], which also provides an extensive suite of tools to explore chemical reaction networks. This makes it desirable to convert reaction rules that are provided in other formats to DPO transformation rules.

The Imaginary Transition State (ITS) graph [15] was introduced as a concise representation of chemical reactions. It is obtained by identifying corresponding atoms in reactants and products according to an atom-to-atom map, inserting the edges of both reactant and product graphs, and attaching special labels to edges that are inserted, deleted, or that change their label, see also [16]. The same graph appears in literature also as “Condensed Graphs of the Reactions” (CGR), usually in a machine learning context [17, 18]. Since the application of a DPO rule to *G* defines an atom-to-atom map, we can think of the edges that are removed from *G* as $E(G)\setminus E(D)$, while $E(H)\setminus E(D)$ is the set of edges newly inserted into the product; an edge that is both removed and inserted changes its label. Thus, *E*(*D*) is the set of bonds that remain unchanged during the reaction. A span $G\leftarrow D\rightarrow H$ thus uniquely defines an ITS graph $\Upsilon$, and conversely, every ITS graph $\Upsilon$ can be regarded as a DPO graph transformation. The rule $L\leftarrow K\rightarrow R$ can be interpreted analogously and thus has an ITS-like graph representation $\Psi$.The rule graph $\Psi$, in fact, turns out to be a subgraph of the ITS graph $\Upsilon$ that contains at least all edges whose labels change, i.e., in chemical terms, the reaction center. This close connection between DPO rules and ITS graphs suggests to considering the problem of inferring reaction rules as a graph-theoretical problem.

The RCLASS data, however, encode information on the bond changes, and thus on the rule graph as a set of local patterns. The construction of DPO graph transformation rules from KEGG RCLASSes is therefore far from trivial. Because of the equivalence of such transformation rules with a core subgraph of the ITS graphs, this task is closely related to the problem of reconstructing a graph from (some of) its subgraphs. Each RDM code can be translated (as we shall see, not always uniquely) to a pair of subgraphs with a partially defined atom-to-atom map, and thus equivalent to a pattern in the ITS. On the other hand, the unchanged parts of reactant and product molecules, which are not covered by RDM codes, must also appear as subgraphs in the ITS graph. Taken together, therefore, the RCLASS entries for enzyme reactions at least implicitly provide a collection of subgraphs that cover the ITS graph.

Graph reconstruction from subgraphs has a long history. The famous *reconstruction conjecture* [19–22] states that graphs with at least 3 or 4 vertices are uniquely determined by the set or multiset of (vertex or edge-deleted) subgraphs, see e.g. [23] for a recent survey. Despite much progress, the conjecture remains open. Computationally, it has been shown that all graphs with up to 13 vertices are reconstructible [24]. Moreover, the probability that a graph is not reconstructible converges to 0 with increasing number of vertices [25]. More general reconstruction problems have been considered, mostly from a probabilistic point of view. Mossel & Ross [26] asked when a graph is reconstructible from its distance *r* neighborhoods and considered reconstructability with high probability in the Erdős-Rényi random graph model. This “graph shotgun assembly problem” has recently received increasing attention. On the other hand, sufficiently small or sparse subgraphs do not seem to be sufficient. For example, it is in general not possible to reconstruct a graph from the multiset of its spanning trees [27].

In light of these theoretical results, there is no reason to expect that RCLASSes will specify unique ITS-like graphs and thus unique DPO rules in all cases. On the other hand, chemical graphs are fairly small, have small degrees, and the different atom and bond labels can provide additional information. Hence, our task is by no means hopeless already at the outset. As we shall see in this contribution, however, non-uniqueness needs to be handled explicitly in each step, and in general, keeping track of multiple competing scenarios cannot be avoided.

## Theory

**Fig. 1:**
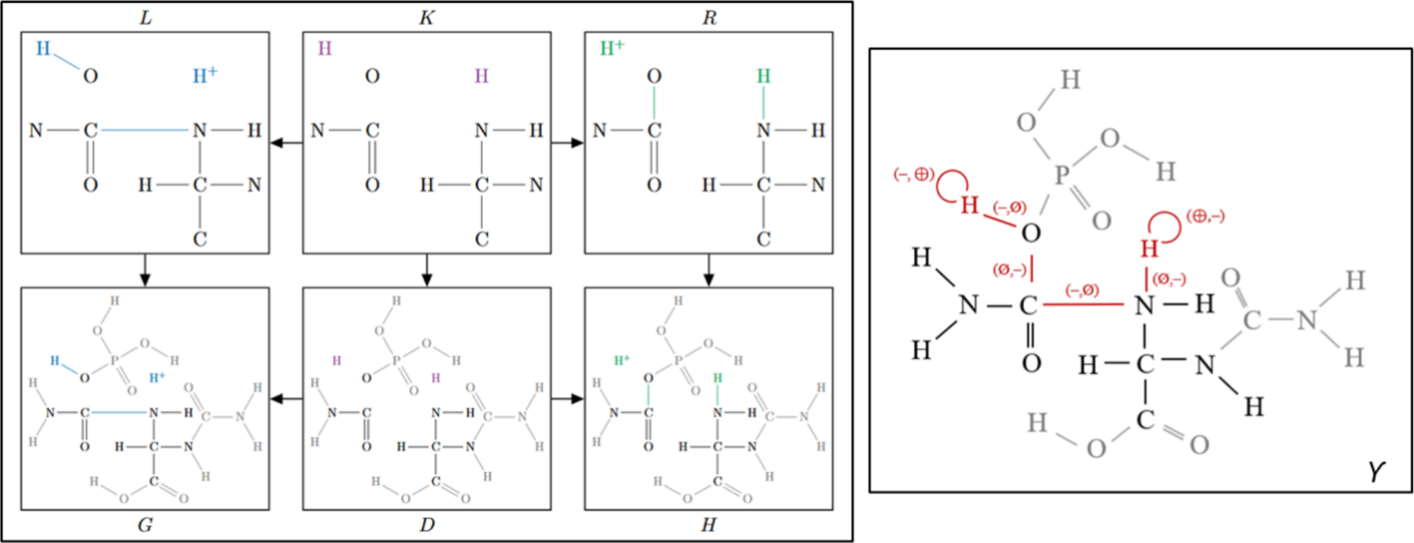
Example DPO rule application (left) and the corresponding ITS graph (right) for the reaction of the enzyme ureidoglycine carbamoyltransferase (R12703)

In this section, we provide a more formal account of DPO graph transformation, its specialization to chemical systems, and, in particular, its close relation with the ITS graph of a reaction and its subgraphs. Although introduced as a formalization of graph transformation, see [11] and the reference therein, the framework is applicable in a wider class of so-called adhesive categories [28, 29]. For our purposes, however, it is sufficient to consider the category of finite labeled simple graphs and graph homomorphisms. A *rule* is a span $L\overset{l}{\longleftarrow }\ K\overset{r}{\longrightarrow }\ R$ of labeled graphs where each arrow denotes a morphism (Fig. 1 left). Intuitively, the graph *L* specifies a pattern that, when matched, is replaced by the graph *R*. The context *K* defines the parts of the pattern *L* that remain unchanged when *L* is replaced by *R*, and thus defines how *R* is inserted where *L* is removed. The application of such a rule to a graph *G* thus requires a match of *L* in *G* that is again specified by a graph morphism $L\overset{m}{\longrightarrow }\ G$. In *G*, first the parts of *L* that are not preserved in *K* are removed, and then the parts of *R* missing in *K* are glued in. More formally, the first of these two steps constructs the graph *D* as a push-out complement for $K\overset{l}{\longrightarrow }\ L\overset{m}{\longrightarrow }\ G$. This graph *D* exists and is unique if *m* satisfies the so-called gluing condition [11]. The second step then constructs *H* as a push-out of $D\leftarrow K\rightarrow R$. We thus arrive at the following commuting diagram for the rule application:

1 $$\begin{aligned} \begin{matrix} L &  \overset{l}{\longleftarrow }\ &  K &  \overset{r}{\longrightarrow }\ &  R \\ \!\!\!\!\!{_m}\downarrow &  &  \downarrow &  &  \downarrow \\ G &  \overset{g}{\longleftarrow }\ &  D &  \overset{h}{\longrightarrow }\ &  H \end{matrix} \end{aligned}$$

The existence of this diagram is thus not guaranteed just by the rule span $L\leftarrow K\rightarrow R$, but depends on finding a match morphism *m* that fulfills the gluing condition. Depending on the choice of the underlying category of graphs, the gluing condition, or parts of it, might be trivially fulfilled. We shall see below that this is indeed the case for chemical rules.

The general framework of DPO graph transformations is much more general than what is needed to model chemical reactions. Here, we consider molecules as vertex and edge labeled graphs and write *V*(*G*) and *E*(*G*) for the vertex and edge sets of a graph *G*. Vertex labels define the chemical elements, and edge labels designate chemical bond types. Thus, graphs do not have parallel edges. For the sake of presentation, we annotate properties such as charges or unpaired electrons on loops, i.e., edges whose endpoints coincide with the same vertex, to avoid describing changes of vertex labels. A chemical reaction is a transformation between molecules that preserves the atoms. It thus can be described by a pair of graphs *G* and *H* whose connected components $A_1,A_2,\dots$ and $B_1,B_2,\dots$ describe the reactant and product molecules, respectively, together with a bijective map $\alpha :V(G)\rightarrow V(H)$ that preserves the vertex labels. The map $\alpha$ is usually referred to as the *atom-to-atom map* (AAM) of the reaction.

Restricting DPO graph transformation to this model of chemical reactions affects the properties of rules and simplifies their application, see [12]. Since chemical reactions preserve atoms, reaction rules describe changes in chemical bonds. Chemical DPO transformations, therefore, are restricted to the deletion, insertion, and relabeling of edges. As a consequence, any match morphism will satisfy the gluing condition. Furthermore, the restrictions $l_V$, $r_V$, $g_V$, $h_V$ of the graph morphisms *l*, *r*, *g*, and *h* to the vertices must be label-preserving bijections that define the AAM for the reaction of *G* to *H* as $\alpha :V(G)\rightarrow V(H)$, $x\mapsto h_V(g_V^{-1}(x))$. The rule itself also defines a bijection between its atoms: $\beta :V(L)\rightarrow V(R)$, $x\mapsto r_V(l_V^{-1}(x))$. Since we are operating in the category of simple labeled graphs (with loops), all graph morphisms preserve edge labels. In particular, therefore, *l*, *r*, *g*, *h* are graph monomorphisms. Of course, the atoms of *L* also must map injectively to the atoms of *G*, hence *m* is also a label-preserving graph monomorphism. The push-out constructions in Eq. (1) then imply that $d:K\rightarrow D$ and $n:R\rightarrow H$ are also label-preserving graph monomorphisms. It will be important below to note that graph monomorphisms are equivalent to (non-injective) subgraph isomorphisms”. Thus, $q:A\rightarrow B$ is a graph monomorphism if and only if *A* is isomorphic to a subgraph of *B*, and *q* defines an embedding of *A* into *B*.

The simple structure of DPO transformation in the realm of chemistry leads to drastically simplified conditions that need to be satisfied for a rule to be applicable to a given target graph *G* [12]. Consider to two distinct vertices $x,y\in V(K)$ such that $r(x)r(y)\in E(R)$ and $l(x)l(y)\notin E(L)$. Note that therefore $xy\notin E(K)$. Suppose $m:L\rightarrow G$ is a match such that $m(l(x))m(l(y))\in E(G)$. Then the rule application tries to create a new edge $n(r(x))n(r(y))\in E(H)$ while the construction of the push-out complement retains the edge $m(l(x))m(l(y))\in E(G)$ as $g^{-1}(m(l(x)))g^{-1}(m(l(y)))\in E(D)$. Thus, *H* winds up with two distinct edges *n*(*r*(*x*))*n*(*r*(*y*)) and

$h(g^{-1}(m(l(x)))h(g^{-1}(m(l(y))))$ connecting the same vertices. This is not in the category of labeled simple graphs. Hence, some of the required push-outs do not exist in this case. Since all chemical transformation operates on edges, leaving the incident vertices unchanged, it is not difficult to check that no other configuration of edges or non-edges between $x,y\in V(K)$, $g(x),g(y)\in V(L)$, $m(g(x)),m(g(y))\in V(G)$, and $h(x),h(y)\in V(R)$ can cause a conflict that prevents the rule application [12]. One way of handling this issue in practice is to associate rules with negative application conditions^1^ [30].

Let us now turn to the connection between chemical DPO transformation and the ITS graph. To this end, we consider the span $G\overset{g}{\longleftarrow }\ D\overset{h}{\longrightarrow }\ H$ where *g* and *h* are bijective on vertices and *assume* that *D* represents all edges that do not change (w.r.t. to their existence of their labels) between *G* and *H*. Note that *D* is (isomorphic to) a subgraph of both *G* and *H* and thus $G\overset{g}{\longleftarrow } D\overset{h}{\longrightarrow }\ H$ is a well-defined span in our category of graphs. Within the DPO transformation framework, $G\leftarrow D\rightarrow H$ models the chemical reaction with reactant graph *G* and product graph *H*. By construction, we have $xy\in E(D)$ if and only if $g(x)g(y)\in E(G)$ and $h(x)h(y)\in E(H)$ have the same edge label, i.e., bond type. *D* thus described the parts of the molecules that remain unchanged during the reaction. In particular, *D* is a maximal (but not necessarily maximum) common subgraph of the reactant graph *G* and the product graph *H*.

Now construct a graph $\Upsilon$ with vertex set $V(\Upsilon )=V(D)$ and edges $xy\in E(\Upsilon )$ if and only if $g(x)g(y)\in E(G)$ or $h(x)h(y)\in E(H)$. We associated with every edge $xy\in E(\Upsilon )$ a pair of labels (*b*(*g*(*x*)*g*(*y*)), *b*(*h*(*x*)*h*(*y*)), where *b*(*uv*) denotes the label of an edge *uv* in *G* or *H* if that edge exists, while $b(uv)=\varnothing$ denotes the absence of the edge in *G* or *H*, respectively. The resulting labeled graph $\Upsilon$ is the ITS graph of the reaction, since it contains for each pair of atoms, an edge whenever there is a bond in at least one of *G* and *H*. By construction it is entirely defined by *G*, *H*, and the AAM between *G* and *H*, i.e., $\alpha {:}{=}h_V(g^{-1}_V)$. We observe that *D* is the subgraph of $\Upsilon$ comprising only the edges with $b(g(x)(y))=b(h(x)h(y)$, i.e., the chemical bonds that remain unchanged during the reaction.

Now consider *any* subgraph $\Psi '$ of an ITS graph $\Upsilon$ that contains all reaction edges, and let $\Psi$ be an isomorphic copy of $\Psi '$ (Fig. 1, right). Clearly, there is a graph monomorphism, i.e., a subgraph isomorphism $d':\Psi \rightarrow \Upsilon$ such that $\Psi '=d'(\Psi )$. For a more detailed description of the relationship between ITS graphs and their subgraphs, we refer to [31, 32]. The main technical result of this section is the following:

### Proposition 1

Given a chemical reaction with reactant graph *G*, product graph *H*, and AAM $\alpha$, or equivalently, the ITS graph $\Upsilon =\Upsilon (G,H,\alpha )$ for this reaction, any subgraph $\Psi$ of $\Upsilon$ that covers all reaction edges defines a unique chemical DPO graph transformation rule $L\leftarrow K\rightarrow R$ that can be applied to *G* and produces *H* as a valid result of transformation.

### Proof

Our goal is to show that (up to isomorphism), $\Psi$ defines a unique DPO rule for the reaction described by $\Upsilon$. To this end, we first note that from the ITS graph $\Upsilon$, we obtain the graphs *G*, *H*, and *D* by deleting from $\Upsilon$ all edges with label $(b_1(e),\varnothing )$, $\varnothing ,b_2(e)$, and $(b_1(e)\ne b_2)$ and retaining as a label only the first or the second component of each label pair, respectively. Since these graphs are defined on the same vertex set $V(\Upsilon )$, we also see that the identity on the vertices defines graph monomorphisms $g:D\rightarrow G$ and $h:D\rightarrow H$. The same construction can be performed for graph $\Psi$ to obtain graphs *L*, *K*, and *R* defined on the common vertex set $V(\Psi )$ with graph monomorphisms $l:K\rightarrow L$ and $r:K\rightarrow R$ that reduce to the identity on the vertices. By construction, the map $d':V(\Psi )\rightarrow V(\Upsilon )$ induces subgraph isomorphisms $m:L\rightarrow G$, $d:K\rightarrow D$, and $n:R\rightarrow H$. Moreover, the AAM $\beta$ on $\Psi$ gives rise to a partial AAM $\beta '=d'(\beta (d'^{-1}))$ on $V(\Upsilon )$ that satisfies $\beta '(x)=\alpha (x)$ for all $x\in d'(V(\Psi ))$. That is, $\alpha$ is a proper extension of $\beta '$ in the sense of [32].

One easily checks, furthermore, that the rule $L\leftarrow K\rightarrow R$ derived from $\Psi$ yields a valid DPO transformation rule when applied to *G* using match *m*. For this purpose, it suffices to consider a pair of vertices $u,v\in V(\Upsilon )$ with $b(g(u)g(v))\ne \emptyset$, $b(h(u)h(v))\ne \emptyset$, and $b(g(u)g(v))\ne b(h(u)h(v))$, since in all other cases, no contradiction can arise [12]. Moreover, *uv* is by definition a reaction edge in $\Upsilon$. By construction of *D*, *uv* is not an edge in *D*, and thus $d^{-1}(u)d^{-1}(v)$ is not an edge in *K*, while $d^{-1}(g(u))d^{-1}(g(v))\in E(L)$ and $d^{-1}(g(u))d^{-1}(g(v))\in E(R)$ are, since $d^{-1}(uv)$ is by assumption contain in $\Psi$. The rule $L\leftarrow K\rightarrow R$ thus explicitly requires the existence of the edge *g*(*u*)*g*(*v*) and specifies that this edge is deleted in the push out complement before *h*(*u*)*h*(*v*) is inserted. Hence, $L\leftarrow K\rightarrow R$ applied to *G* with the subgraph isomorphism *m* correctly produces both the push out complement *D* and the push out *H*. $\square$

In less formal terms, Prop. 1 ensures that, given a reaction with ITS graph $\Upsilon$, there are DPO rules that “explain” the reaction: in fact, *any* subgraph of the ITS graph that contains the reaction center can be chosen for this purpose. Note that Prop. 1 only guarantees the existence of at least one matching morphism $m:L\rightarrow G$ with this property. There may be alternative embeddings of *L* in *G*, some of which may lead to a violation of the gluing condition and/or produce reaction products $H'$ not isomorphic to *H*.

There is a strong correspondence between the rule spans and subgraphs of ITS graphs, even though there is no one-to-one correspondence. The reason is that for fixed *L*, *R*, and bijection $\beta :V(L)\rightarrow V(R)$, the context graph *K* is not unique. There is, however, a unique maximal context graph $\hat{K}$. To see this, first note that by assumption, there are bijective maps $g_V:V(K)\rightarrow V(G)$ and $h_V:V(K)\rightarrow V(H)$ for any choice of *K*. Moreover, $g_V$ and $h_V$ extend to graph monomorphisms whenever $g:K\rightarrow G$ and $h:K\rightarrow H$ if and only if $xy\in E(K)$ implies $g(x)g(y)\in E(G)$, $b(xy)=b(g(x)g(y))$, $h(x)h(y)\in E(H)$, $b(xy)=b(h(x)h(y))$. Denote by $\hat{K}$ the graph defined by the converse implication, i.e., $xy\in E(\hat{K})$ if and only if $g(x)g(y)\in E(G)$, $h(x)h(y)\in E(H)$, and $b(g(x)g(y))=b(h(x)h(y)){=}{:}b(xy)$. We see that *g* and *h* are graph monomorphisms for every spanning subgraph *K* of $\hat{K}$. On the other hand, $\hat{K}$ is exactly the context graph that is obtained by the construction of rules from the ITS graphs above. It is not difficult to verify that for given *G*, *L*, *R*, $\alpha =h_V(g_V^{-1})$, and matching monomorphism $m:L\rightarrow G$, the rules $L\overset{g}{\longleftarrow }K\overset{h}{\longrightarrow }R$ and $K\overset{g}{\longleftarrow }{\hat{K}}\overset{h}{\longrightarrow }R$ yield the same graph *H*. For given *R*, *L*, and atom map $\beta : V(L)\rightarrow V(R)$, it is therefore sufficient to consider the rule with maximal context $\hat{K}$. For any other choices of *K*, some edges are deleted and re-inserted with the same label. Clearly, this is equivalent to propagating the edges from *L* to *R* without change, and hence does not produce a different graph transformation.

**Fig. 2:**
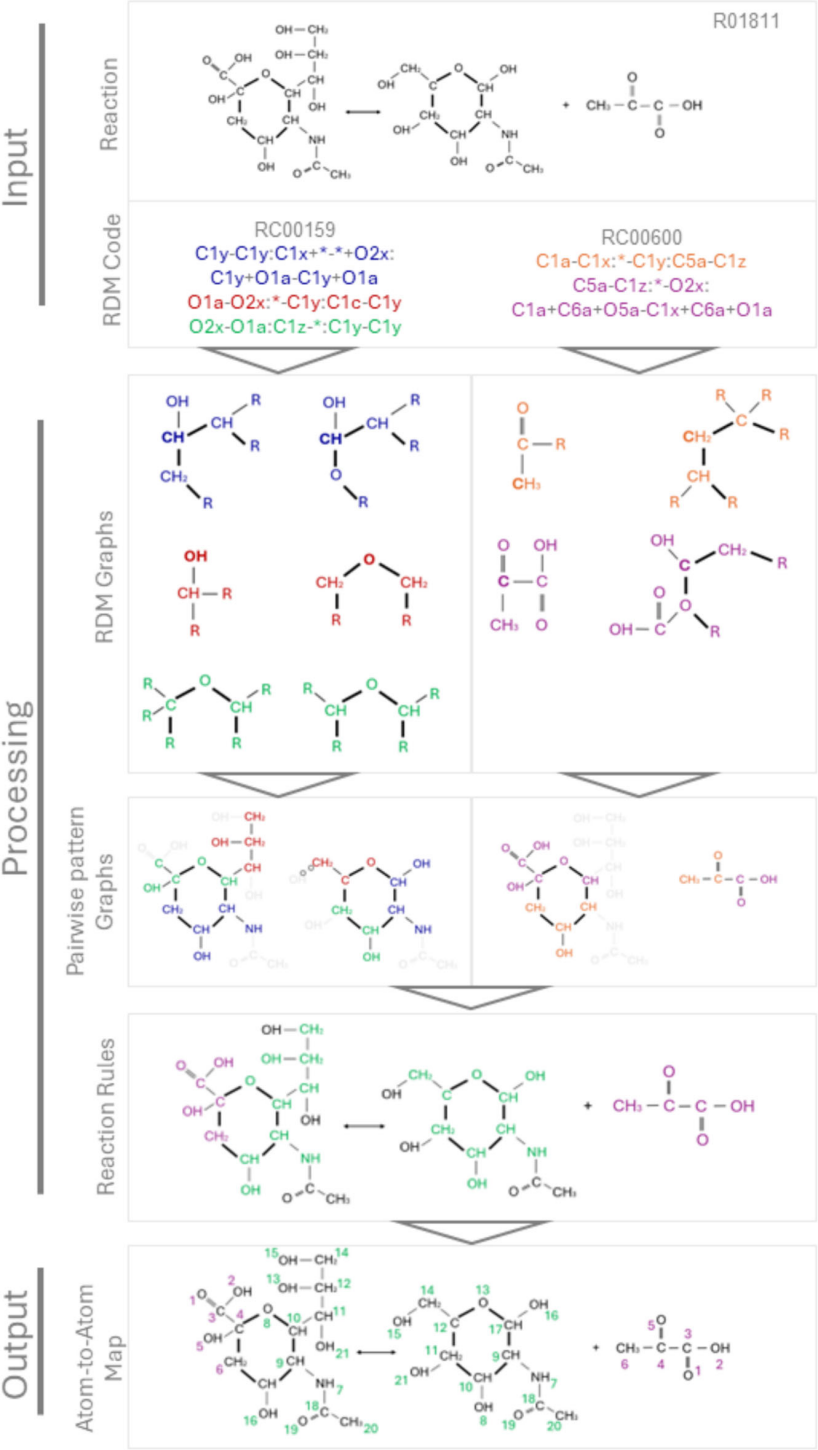
Overview of the workflow shown with the example reaction R01811 (N-acetylneuraminate pyruvate to N-acetyl-D-mannosamine and pyruvate). Two RCLASSes with respective RDM codes are assigned to this reaction (RC0159 and RC00600). After processing by the laveau tool, an atom-to-atom map, as shown below, is generated

## Methods

### Workflow overview

The generation of atom-to-atom reaction maps from RDM codes in the KEGG database involves several steps. These steps are summarized in Fig. 2 for an overview. First, the KEGG RDM codes are translated into RDM graphs, which are then parsed together with the molecular graphs linked in the database. Each RDM graph defines patterns that link one reactant and one product molecule, and together with the correspondence of some of the atoms in this pair of patterns. The RDM graphs are a faithful translation of the RDM data of an RCLASS into a graph pair with some matching atoms.

The pairwise pattern graphs of individual RCLASSes are then combined into reaction rule graphs by considering all combinations of possible embeddings of the RDM graphs patterns into the molecules of associated with the RCLASS. Using the correspondences between atoms in the RDM pattern graphs as constraints, all partial atom-atom maps between the molecules are enumerated. The result of this step is a set of *pairwise pattern graphs* that translate the information of individual RCLASSes into pairs of subgraphs, one each of a substrate and a product molecule, together with the atom-atom correspondences between them.

For each KEGG reaction, we combine the *pairwise pattern graphs* of all associated RCLASSes, again exhaustively enumerating all possible combinations. This results in reaction rule graphs that cover the reaction center, provided that RCLASSes are available for all relevant pairs or molecules. In this case, we obtain a complete DPO rule graph that covers all bond changes during the reaction, together with an atom-to-atom mapping for the part of the molecules covered by the RCLASS data. Otherwise, the DPO rule is incomplete. In either case, we consider all applications of the rule to the KEGG reactions. In the final step, a complete atom-to-atom map is computed for a given KEGG reaction using the embedding of the DPO rule in the reactant and product graphs as a constraint. The final atom-to-atom map is computed as an extension using the partial atom-to-atom map defined by the reaction rule graphs.

Our workflow is designed to collect, in each step, all possible solutions that are consistent with the raw RCLASS data, i.e., we exhaustively enumerate all atom-to-atom mappings that are consistent with the provided RCLASS data. For each KEGG reaction, we therefore obtain one or more AAMs and thus equivalent ITS graphs. Each of these is, by construction, consistent with the curated RCLASS data for that reaction. Together with the ITS graph $\Upsilon$, we also construct a subgraph $\Psi$ of $\Upsilon$ that contains the atoms and bonds specified by the RDM codes associated with relevant RCLASSes, as well as all reaction edges and reaction vertices defined in the ITS graph $\Upsilon$. Hence, $\Psi$ and $\Upsilon$ satisfy the assumptions of Prop. 1, and therefore $\Psi$ is equivalent to a DPO rule that—when applied with the correct subgraph match of *L* into reactant graph *G*—correctly yields the product graph *H*.

For some reactions, we indeed obtain more than one reaction rule and more than one complete atom-atom map. As we shall see in the evaluation section below, however, such residual ambiguities are rare, and for the overwhelming majority of the reaction data, a single rule and a single atom-to-atom map can be derived. We also encounter some cases where no solution is found, i.e., where the RDM annotation results in conflicts that leave no choice of a consistent AAM.

The goal of this work is to convert the RCLASS information associated with KEGG reactions into explicit AAMs for the reactions and associated DPO rules that capture the RCLASS data themselves. Therefore, we made no attempts to resolve ambiguities or conflicts since this would require altering, ignoring, or amending some of the information provided by the KEGG database.

### KEGG RCLASSes

In the following, we write $G'\subseteq G$ if $G'$ is a subgraph of *G*, where $G'$ inherits all vertex and edge labels from *G*. Moreover, it will be convenient to think of the reaction center as a pair of (not necessarily induced) subgraphs $L\subseteq G$ and $R \subseteq H$ such that (i) the restriction of the atom-to-atom map to *R* and *L*, i.e., $\alpha :V(L)\rightarrow V(R)$, is again a bijection and (ii) *L* and *R* contain all vertices incident with bond changes. We do not assume that *L* and *R* are minimal with this property, i.e., *R* and *L* may contain some common unchanged “context”.

**Fig. 3:**
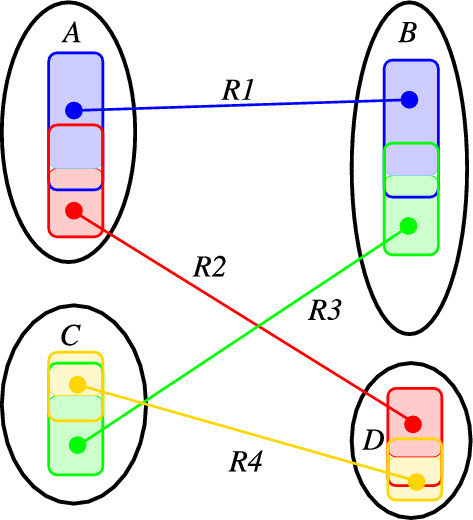
Reaction $A+C\rightarrow B+D$ gives rise to four RCLASSes, one for each combination of a reactant with a product (indicated by different colors). Each RCLASS $\mathcal {R}_{ij}$ implicitly defines a pair of subgraphs $\widehat{L}_{ij}$ and $\widehat{L}_{ij}$ (shown by bold colored outlines) that contain subgraphs $L_{ij}$ and $R_{ij}$ (shown by the shaded areas). The latter are linked by a 1-1 of their atoms. Together, the unions of the subgraphs  and  exhibit a 1-1 correspondence of their vertices that corresponds to a DPO graph transformation rule

In KEGG RCLASSes, the AAM $\alpha :V(L)\rightarrow V(R)$ is split into partial maps between pairs of molecules (Fig. 3). For each pair $(A_i,B_j)$ of molecules, one considers subgraphs $L_{ij}\subseteq \widehat{L}_{ij}\subseteq L_i\subseteq A_i\subseteq G$ and $R_{ij}\subseteq \widehat{R}_{ij}\subseteq R_j\subseteq B_j\subseteq H$ such that the restriction $\alpha _{ij}:V(L_{ij})\rightarrow V(R_{ij})$ of $\alpha$ is a bijection. The vertices in $V(\widehat{L_{ij}})\setminus V(L_{ij})$ and $V(\widehat{R}_{ij})\setminus V(R_{ij})$ are not mapped between type graphs $A_i$ and $B_j$. In other words, $x\in V(\widehat{L}_{ij}){\setminus } V(L_{ij})$ implies $\alpha (x)\notin B_j$ and $y\in V(\widehat{R}_{ij}){\setminus } V(R_{ij})$ implies $\alpha ^{-1}(y)\notin A_i$. Since $\alpha :V(G)\rightarrow V(H)$ is a bijection, for each $x\in V(\widehat{L}_{ij})\setminus V(L_{ij})$ there is a $B_k$, $k\ne j$, such that $\alpha (x)\in V(B_k)$ and, for each $y\in V(\widehat{R}_{ij}\setminus V(R_{ij})$ there is a $A_h$, $h\ne i$ such that $\alpha ^{-1}(y)\in V(A_h)$.

The data describing a KEGG RCLASS consists, implicitly, of $L'_{ij}\subseteq \widehat{L}'_{ij}$ and $R'_{ij}\subseteq \widehat{R}'_{ij}$ together with an atom-to-atom map $\beta _{ij}:V(L'_{ij})\rightarrow V(R'_{ij})$ and the molecular graphs $A_i$ and $B_j$. Considering all RCLASSes belonging to a given reaction, the task is therefore to reconstruct the subgraphs $L\subseteq G$ and $R\subseteq H$ satisfying $V(L)=\bigcup _{i,j} V(\widehat{L}_{ij})$ and $V(R)=\bigcup _{i,j} V(\widehat{R}_{ij})$ together with the atom-to-atom map $\alpha :V(L)\rightarrow V(R)$ such that (i) there are subgraphs $\widehat{L}_{ij}\subseteq A_i$ with $\widehat{L}_{ij}\simeq \widehat{L}'_{ij}$ and $\widehat{R}_{ij}\subseteq B_j$ with $\widehat{R}_{ij}\simeq \widehat{R}'_{ij}$ and (ii) the restriction of $\alpha :V(L_{ij})\rightarrow V(R_{ij})$ coincides with $\beta :V(L'_{ij})\rightarrow V(R'_{ij})$. In the following, it will be convenient to combine the graphs $\widehat{L}'_{ij}$ and $\widehat{R}'_{ij}$ and to represent the bijection $\beta _{ij}:V(L'_{ij})\rightarrow V(R'_{ij})$ as a bipartite matching $M_{ij}$ between them. We will refer to $({L}'_{ij},\widehat{R}'_{ij},M_{ij})$ collectively as a *pairwise pattern graph*.

### From RDM codes to RDM pattern graphs

**Fig. 4:**
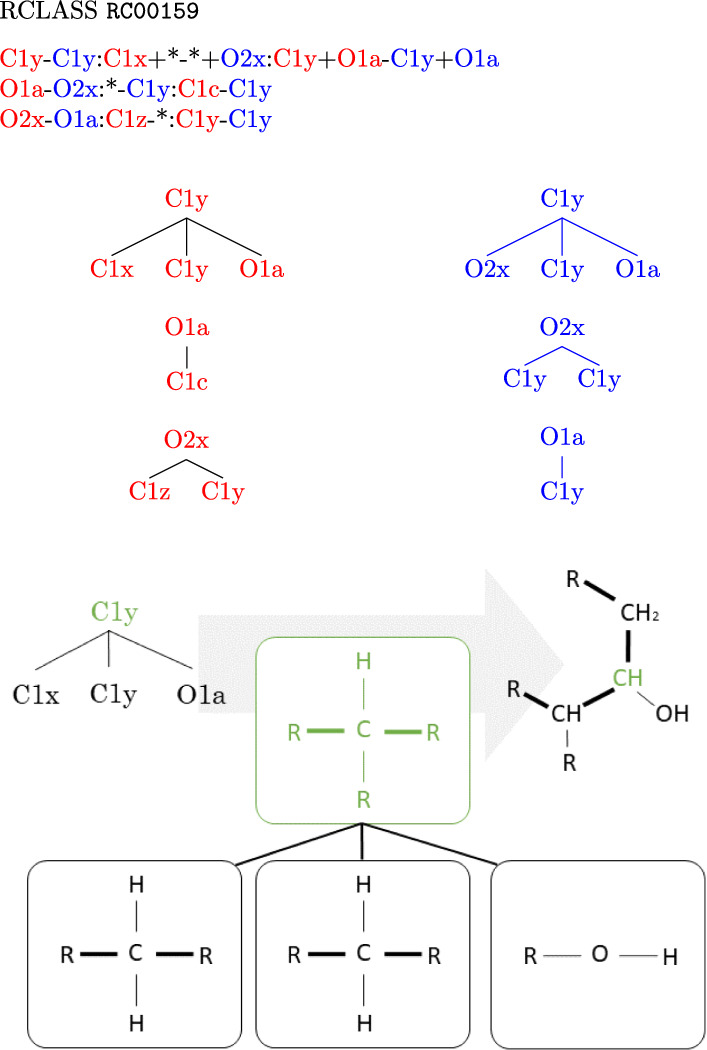
Expansion of RDM codes into pairs of ordered trees. RCLASS RC00159 is specified by three separate RDM codes. Each consists of an R-atom, a D-atom, and M-atoms arranged in this order, separated by colons. Atom types for the reactant and product side (shown here in red and blue) are separated by a hyphen. In the first step, each RDM code is expanded into a tree with the R-atom as the root. In the second step (shown here for the first red tree), the atom types are replaced by their chemical structure. Along each tree edge, a pair of wildcards, i.e., “R-residues”, is replaced by the corresponding chemical bond (accounting for matching bond types)

A major practical complication is that KEGG does not explicitly provide the *pairwise pattern* graphs $({L}'_{ij},\widehat{R}'_{ij},M_{ij})$ for reactant $A_i$ and product $B_j$. Instead, this information is provided implicitly in the form of so-called RDM codes. The RDM codes were introduced in [7] and make use of a list of atom types that depend not only on the atom label but also on the focal atom’s chemical neighborhood [8]. Each of the currently 68 local *type graphs* is assigned a label. The atom type codes distinguish between bonds that are contained in rings, shown in bold, and non-ring bonds (Fig. 4). In addition, single, double, triple, and aromatic bonds (always in rings) are distinguished. For example, the carbon atom in $\text {R}-\text {CH}_{3}$ has type C1a, while the carbon in $\text {R}-\text {CH}_{2}-\text {R}$ has type C1b.

The definition of an RDM code for a pair of reactant and product molecules distinguishes two situations:

(i) If the reactant/product pair has unmatched non-hydrogen atoms, then the R-atom represents the atom that belongs to the matched structure and is adjacent to an unmatched atom. A D-atom is then an unmatched atom adjacent to the R-atom, while M-atoms are matched atoms adjacent to the R-atom.
(ii) If there are no unmatched non-hydrogen atoms, then the R-atom is a (matched) atom whose type changes in the reaction and whose adjacent atoms have no type change.

In general, multiple RDM codes are given for each reactant/product pair. Multiple D-atoms or M-atoms are specified by using + as a separator in the corresponding section. An asterisk is used to denote the absence of non-hydrogen atoms.

The translation of these RDM codes into pattern graphs proceeds in two stages. First, the RDM codes are separated into reactant and product parts, which are separately transformed into a tree, each with the R-atom as the root (Fig. 4).

The correspondence between atoms specified in the RDM code and also the classification of atoms as R- M- and D-atoms are recorded. In the second step, the atom types are replaced by their type graph representation as defined in [7, 8]. Since all other atoms in the RDM code are by definition adjacent to the R-atom, each tree corresponds to a connected graph, derived by merging adjacent type graphs progressively to the root. If two *type graphs* have a common pair of atoms, they serve as the merge-point. For instance, O1c (H–O–P) and P1b (P–O) share the P–O motif and are merged to H–O–P. If no such pair can be found, *wildcard residues*, R, which designate non-hydrogen atoms, must be used. The merging along an edge must respect the bond type and, in most cases, constitutes the replacement of a wildcard R by the adjacent focal atom.

Importantly, there may be more than one distinct solution to merge two type graphs. For example, the merge of two adjacent C2b atom types, R–CH=R, yields either R–HC=CH–R or R=CH–HC=R. Similarly, ambiguous steps appear when wildcards are linked with ring and non-ring bonds. Non-extensible combinations in general become only recognizable in later merge steps; it is therefore necessary to search all possible merges of two adjacent *type graphs* in a branch-and-bound fashion. In the example above, only the first solution of merging can be extended by C1a, $\text {R}-\text {CH}_{3}$, to yield $\text {R}-\text {HC=CH}-\text {CH}_{3}$.

**Fig. 5:**
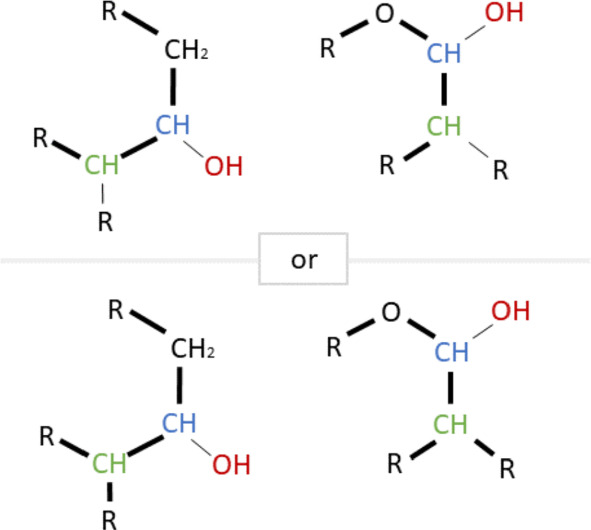
Each RDM code corresponds to a pair of *RDM pattern graphs* connected by a 1 by 1 correspondence on a subset of vertices, shown here by atoms with the same non-black color. Unmatched D-atoms are shown in black. The example corresponds to the first pair RDM trees in Fig. 4. All possibilities for compiling the RDM code are shown

As a final result, *RDM pattern graphs* together with mappings between some of their atoms are obtained. For example, for the first RDM code in Fig. 4, we obtain the following two patterns, where corresponding colors indicate matched atoms, Fig. 5.

Unfortunately, even this relatively simple first step does not always yield an unambiguous pattern. The KEGG database indicates alternatives for five graph representations (C2y, N1a, N1b, N1c, N2a) [8]. However, there are an additional 20 cases where alternative interpretations of RDM codes need to be taken into account. Moreover, some symbols require up to four distinct alternatives. For example, P1a may correspond to the following *type graphs*: P–R, R–P(–R)(–R)(=R), R–P(–O)(=R), and R–ring–P–ring–R. An additional complication arises from the fact that within-ring bonds may also be interpreted as aromatic bonds, and *vice versa*. Furthermore, bonds involving O do not distinguish between single and double bonds. Changes in atomic charges can also result in different patterns being represented by the same RDM code. A complete list of all representations of RDM codes can be found in the Appendix. As a consequence, the reconstruction of the *RDM pattern graphs* is not unique. For the example in Fig. 4, all possible *RDM pattern graphs* are shown in Fig. 5. All such alternatives are carried forward to the next analysis step.

### Pairwise pattern graphs: embedding RDM pattern graphs into molecule graphs

**Fig. 6:**
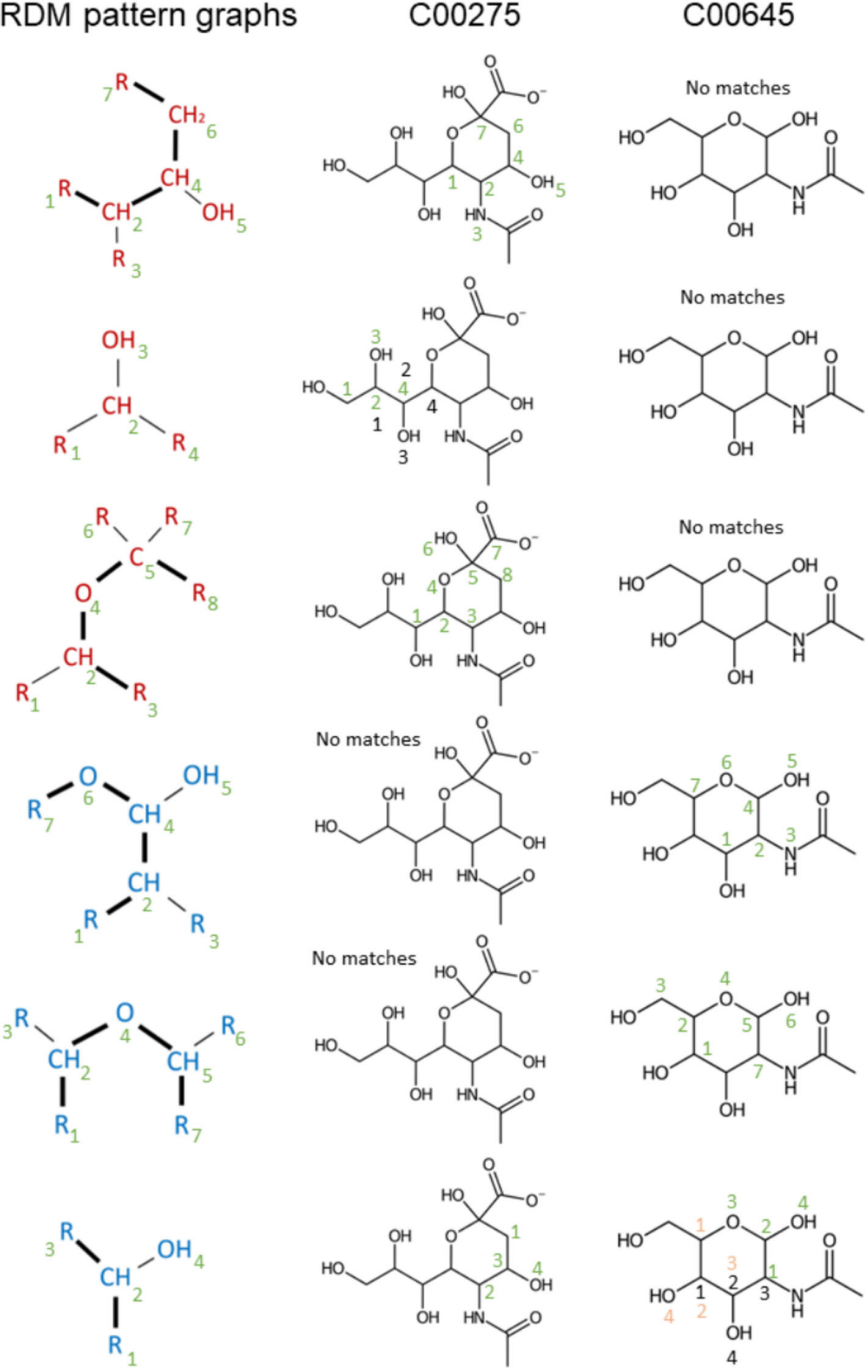
Mapping of *RDM pattern graphs* to a pair of reactant/product graphs. Numbers indicate matches between atoms of patterns and molecules. Where alternative embeddings are possible, they are shown in different colors. Here, the left-hand side of the RDM codes matches C00275 (mannose 6-phosphate) and the right-hand side matches C00645 (N-Acetyl-mannosamine). Note that the matches of the *RDM pattern graphs* partially overlap

In the next stage, a *pairwise pattern graph* is obtained by merging constituent *RDM pattern graphs*. Recall that the *RDM pattern graphs* of an RCLASS are isomorphic to subgraphs of the reactant or product molecules. Thus, all subgraph embeddings are considered. Since KEGG stores the *RDM pattern graphs* without an explicit distinction of reactant and product side, these embeddings are first used to determine the molecules corresponding to the left and the right part of the RDM codes. In general, the embedding of an *RDM pattern graph* into a molecule is not unique (Fig. 6). Moreover, the embeddings may overlap partially.

**Fig. 7:**
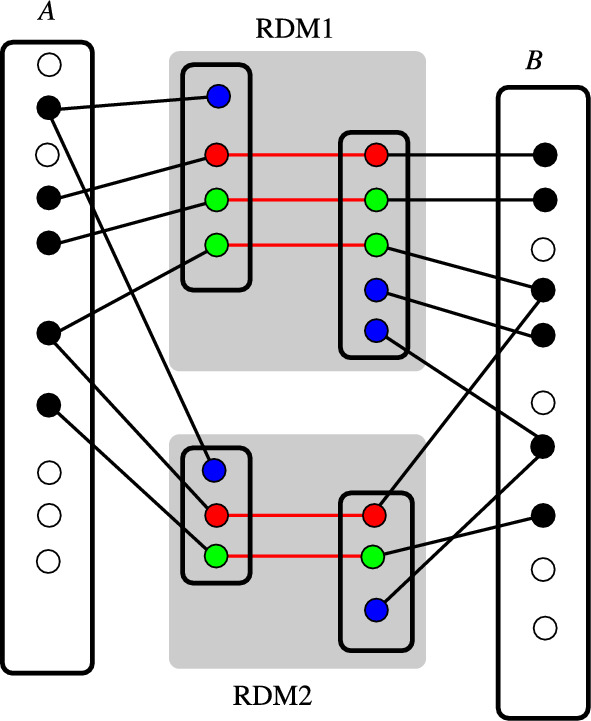
Consistency of overlapping *RDM pattern graphs*. The vertex sets of two RDM patterns (gray background) are shown with respective R-atoms in red, D-atoms in blue, and M-atoms in green. Red lines indicate matches between reactant and product patterns. Black edges indicate the embeddings into the reactant *A* and the product *B*. D-atoms are embedded on one side only, while R-atoms and M-atoms have corresponding vertices in *A* and *B*. Whenever in the embedding of the left-hand side two RDM patterns overlap in *A*, either the vertex is a D-atom in both RDM patterns, or the matching atoms on the right-hand side of the RDM patterns also overlap in the embedding into the product side *B*. Both *A* and *B* may contain atoms that do not appear in any RDM pattern

Overlapping embeddings of *RDM pattern graphs* must be consistent in the following sense: Suppose the embeddings of the left-hand sides of two *RDM pattern graphs* share a vertex *v* of *A*, and denote $x_1$ and $x_2$ by the corresponding vertices in the *RDM pattern graphs* (Fig. 7). Then either both $x_1$ and $x_2$ are D-vertices, or both are matched to atoms $y_1$ and $y_2$ on the right-hand side of the corresponding patterns. In the latter case, the embeddings of the right-hand sides into *B* must be such that both $y_1$ and $y_2$ are mapped to the same vertex *w* in *B*. Then *v*-*w* becomes an edge in the matching obtained from patterns obtained form by the union of the two RDM pattern graphs. An analogous condition holds if embeddings of the right-hand sides of two *RDM pattern graphs* share a vertex. Given the embeddings, this condition can be easily checked. In the case of multiple embeddings, all combinations have to be checked at all overlapping vertices, and combinations that violate these consistency conditions are rejected.

From this section, result one or more pairs of embeddings of pattern graphs $\widehat{L}_A$ and $\widehat{R}_B$ into *A* and *B* together with a matching $M_{AB}$ between their vertex sets, i.e., a *pairwise pattern graph*. Note that non-hydrogen atoms that remain unmatched between $L_A$ and $L_B$ are necessarily D-atoms in some RDM code. Thus, these atoms appear only in either the reactant *A* or the product *B*. Since, by definition of the RDM codes, the bond between an R-atom and a D-atom is broken in the reaction, the D-atoms are part of the reaction center. They will appear as matched atoms in the *pairwise pattern graph* for another reactant/product combination. For atoms that occur in two RDM codes, information on the atom type (R, D, or M) and the atom-to-atom map from both sources is recorded at these nodes. This will be required for further processing, as we shall see in the next section.

### Reconstruction of reaction rules

Most reactions involve more than one reactant or product. In these cases, the reaction gives rise to several different RCLASSes, one for each reactant/product pair that exchanges atoms. For each of them, we obtain pairs of patterns together with feasible embeddings and corresponding matchings of reactant and product atoms. Suppose the RCLASS $\mathcal {R}$ linking *A* and *B* has a D-atom (w.r.t. one of the RDM code of $\mathcal {R}$), this is mapped to the vertex $x\in V(A)$. Then, there must be a second product molecule $B'$ and an RCLASS $\mathcal {R}'$ linking *A* and $B'$ such that *x* is an R-atom or an M-atom in some of the RDM code of $\mathcal {R}'$, and thus *x* must have a matching vertex in the product molecule *B*.

As already indicated in the previous section, some atoms have multiple assignments of the atom type label. The different possible atom types must be tested individually in most cases because there is no general rule on how the atom types should be evaluated. The only exceptions are atoms with an R-atom label. Regardless of the other atom type labels, these are always classified as R atoms. All atom labeled M and D will be treated first as M-atoms and then as D-atoms. Incorrectly declared M-atoms are later filtered out by a balance test of the atom types, and incorrect D-atom notations are filtered out by testing the DPO rule.

The *pairwise pattern graphs* as embedded into their corresponding reactants and products $A_i$ and $B_j$ thus can be combined in such a way that their union determines (1) a matching between *V*(*G*) and *V*(*H*), and (2) every vertex in *V*(*G*) in the embedding of $\widehat{L}_{ij}$ but remains unmatched in $M_{ij}$ appear in some other $\widehat{L}_{hk}$ as a matched vertex. Analogously, every vertex in *V*(*H*) in the embedding of $\widehat{R}_{ij}$, which is also remains unmatched in $M_{ij}$, appears in some other $\widehat{R}_{hk}$ as a matched vertex. The union of the embeddings of all *pairwise pattern graphs* $(\widehat{L}_{ij},\widehat{R}_{ij},M_{ij})$, therefore, defines subgraphs $L\subseteq G$ and $R\subseteq H$. Since the embeddings of the pairwise pattern graphs must be consistent with a single reaction, the union $M{:}{=}\bigcup _{ij} M_{ij}$ of the bipartite matchings must again be a bipartite matching. Moreover, since every vertex in *V*(*L*) and *V*(*R*) is contained in one of the partial matchings $M_{ij}$, the union matching *M* defines a bijection $\alpha _M:V(L)\rightarrow V(R)$ by setting $\alpha (x)=y$ if and only if $xy\in M$. The union of the matchings between the union of pattern graphs, therefore, defines the atom-to-atom map between *L* and *R*. The graphs *L*, *R*, and the map $\alpha :V(L)\rightarrow V(R)$ also define a graph *K* with vertex set $V(K)=V(L)$ and edges $xx'\in E(K)$ if and only if $xx'\in E(L)$, $\alpha (x)\alpha (x')\in E(R)$, and the edges $xx'$ and $\alpha (x)\alpha (x')$ have the same label. Then $L\leftarrow K\rightarrow R$ with the subgraph isomorphism specified by the identity between *K* and *L*, and $\alpha :V(K)\rightarrow V(R)$ is the desired DPO transformation rule. Note that, since *L* and *R* are derived from a consistent embedding of $(\widehat{L}_{ij},\widehat{R}_{ij},M_{ij})$ into the reactant and product graphs *G* and *H*, the rule $L\leftarrow K\rightarrow R$ by construction provides a mathematically valid description of the reaction between *G* and *H*, i.e., the diagram (1) necessarily commutes.

Again, we cannot assume that embeddings of $L_{ij}$ and $R_{ij}$ into $A_i$ and $B_j$ are unique. If more than one embedding exists, all combinations must be tested for conditions (1) and (2) above. In addition, a particular *RDM pattern graph* can also be used several times to describe a reaction. Therefore, all combinations must also be included and tested here. Conversely, if no combination of embeddings exists such that the union *M* of the matchings is again a matching, then the pairwise pattern graphs are in conflict with each other, and no DPO rule exists that is consistent with the given RDM data. Our workflow, therefore, exhaustively enumerates all DPO rules (up to left-out edges in *K*) and their embeddings into *G* and *H* that are consistent with the KEGG RCLASS information for a given reaction.

### Missing RCLASSes

For some reactions with multiple reactants and products, KEGG does not provide RLCASS data for all reactant/product pairs. For example, for R00912,

C00002 + C00062 + C00099 $$\Longleftrightarrow $$C00008 + C00009 + C05340,

only three of the nine possible RCLASSes are listed. While some combinations are empty, C00009 (phosphate) does not appear in any one of them. Such examples violate the assumption that the entire reaction center will be covered by RDM codes. The superposition of the pairwise pattern graphs will leave some D-atoms unmatched in this case. In this situation, it is not possible to extract the atom-to-atom map from the rule directly, since we only have partial ITS graph both at the level of the reactants and products and at the level of their rule subgraphs, see also [31].

**Fig. 8:**
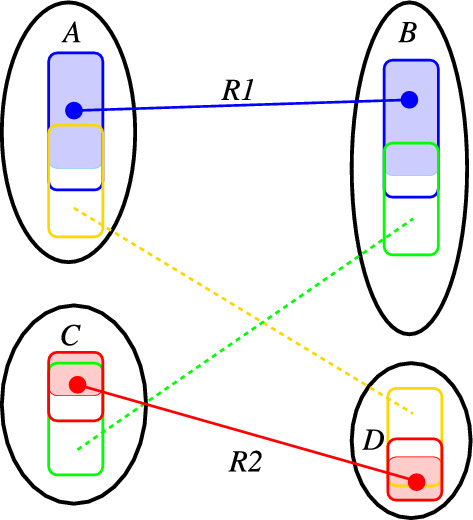
If RCLASSES are missing, not all atoms in the ITS are covered, and the rule is also incomplete. In this example, comprising the two reactants (*A* and *B*) and two products *C* and *D*, four RCLASSes are required to completely describe the reaction. If only R1 and R2 are used, then the green and yellow portions are not represented. As a consequence, the derived DPO is incomplete and does not explain the reaction

One possibility to handle this case is to first construct complete atom-to-atom maps for the reaction as extensions of the partial atom-to-atom maps of the embedded incomplete rule graph. Consider the RCLASS *R*1 in Fig. 8.

It defines a partial bijection between molecules *A* and *B* covering the blue shaded area. The D-atoms of the contributing RDM classes (white area within the blue boundaries) are known to be mapped to other molecules. Since *A* and *B* are molecules, the set of blue atom-to-atom matches extends to a *connected* common subgraph that does not contain any of the (blue) D-atoms. More precisely, the possible matches comprise only the atoms in *A* and *B* that are reachable from an atom in the initial match set without passing through one of the D-atoms. Such an extension of the matches implied by *R*1 could be computed by the VF2 algorithm initialized with the matches defined by *R*1, using only the reduced set of candidate matches, and restricting the result to a “connected” graph (where the set of *R*1 matches is treated as a single connected component).

The remaining, unmatched vertices in *A* and *B* then need to be matched with the products *D* and *C*, respectively, where, in the example of Fig. 3, only the vertices not covered by an analogous extension of *R*2 must be considered. Note that we cannot make the assumption of connectedness here for the missing RCLASSes since we do not know the atom-to-atom matches among which the bonds are modified. We do know, however, that the D-atoms of *R*1 in *A* and *R*2 in *D*, as well as the D-atoms of *R*2 in *C* and *R*1 in *B*, need to be matched onto each other in this example of Fig. 8, thus providing additional constraints.

In general, this procedure may still leave some atoms unmatched that are subject to changes of their chemical environment but are not covered by any of the specified RCLASSes. The resulting partial atom-to-atom map $\beta$ could then be completed using the assignment approach described in [33] as part of an MCS-based atom-to-atom mapping tool. An appealing alternative is to adapt the $A^*$-search algorithm of [34] that minimizes a graph editing distance in such a way that the partial atom-to-atom maps described above can be used as constraints.

Instead, here we use the D-atoms of the available RCLASSes to produce candidate atom-to-atom maps for the reaction center. Therefore, on both reaction sides, connected nodes representing D-atoms are extracted as subgraphs. Then we compute, for each subgraph, a corresponding isomorphic subgraph on the opposite side of the reaction using the VF2 algorithm. A subgraph match only succeeds if the following conditions are satisfied:

1. The match is not one of the original pairwise pattern graphs.
2. If it matches other atoms of an integrated pairwise pattern graph, at least one R-atom must be present.
3. It is only allowed to map to D-nodes from other pairwise pattern graphs if the matching consists exclusively of D-nodes.
4. Hydrogen atoms are generally excluded from the matching process.

**Fig. 9:**
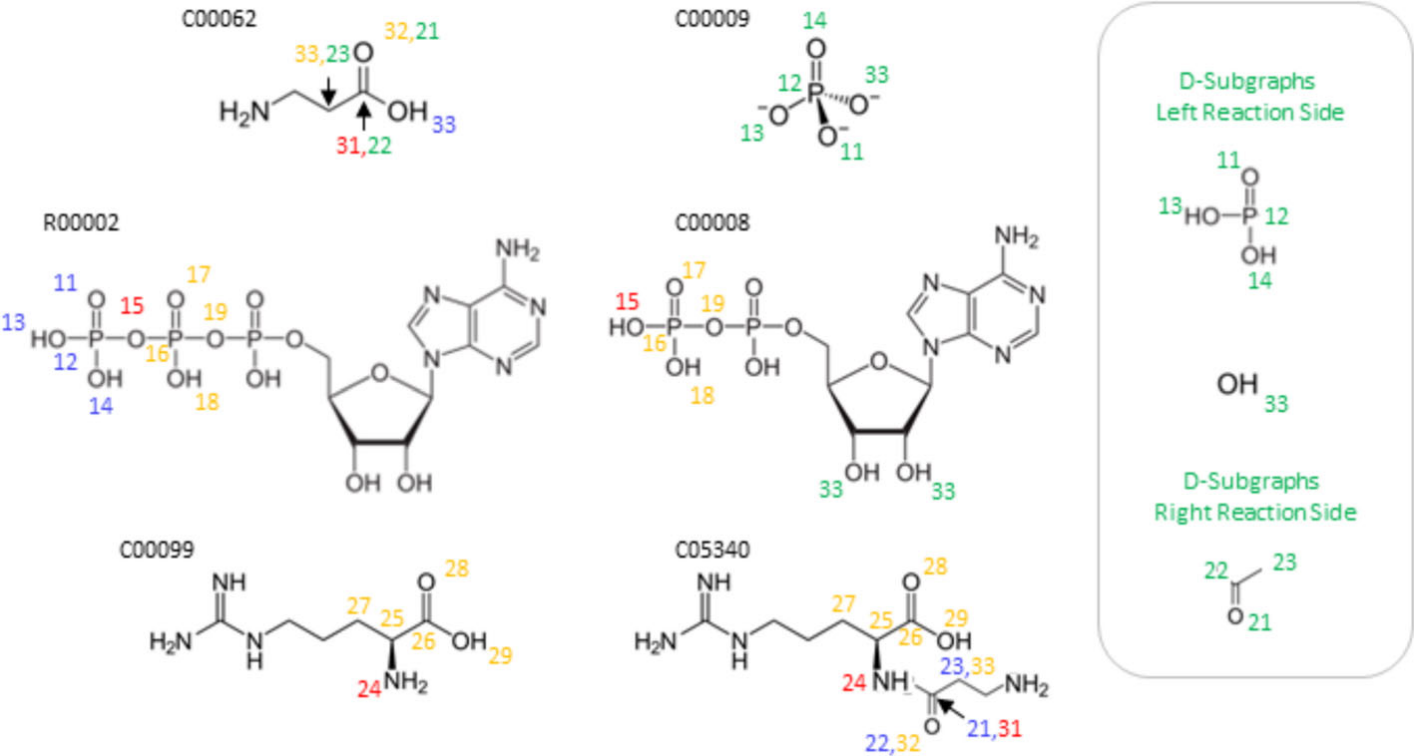
Completion of missing mappings by D-atoms on the example reaction R00912. In this reaction, three RDM codes are assigned. The resulting atom maps can be distinguished by the first digit of their IDs. Red IDs indicate R-atoms, yellow M-atoms, and blue R-atoms. On the right side of the picture are the D-subgraphs that can be extracted from all RDM codes. The green IDs show the identified mapping candidates, which are then checked for relevance by using the DPO rule

Again, we enumerate all extensions meeting these conditions. For example, for reaction R00912 in Fig. 9, a total of three different mapping candidates are constructed.

Since one of the D-subgraphs consists of only one oxygen, the number of candidates increases above average, but only one mapping candidate (O33 mapping to Compound C00009) is plausible for this reaction. To check this automatically, all candidates are first checked for the balance of atom types (R-, D-, and M-atoms). If this is the case, they are then included in the DPO rules. Therefore, the final step is to recover the subgraphs *L* and *R* as the union of the subgraphs defined by existing RCLASSes together with all edges not yet in *R* or *L* that change according to the atom-to-atom map $\beta$ and their incident vertices. Since the RCLASS does not cover transformation rules for most small molecules such as CO2 or H2O, two different errors may occur. First, the matching of D-Atoms can fail if there are bond changes between the D-atoms, as seen in the typical cleavage of carboxy groups ($\text {R-COOH}$) to O=C=O. To address this, a special case was implemented to recognize these instances and match the carboxy groups to CO2. Secondly, if individual atoms of small molecules are added to a molecule (if there are no hydrogens), the matching of D-Atoms may succeed, but the actual reaction rule remains incomplete because structural changes in the rest of the small molecule are not considered. To remedy this, a function was added to complete the reaction rule. A more detailed explanation can be found in the Appendix.

The final check whether the procedure is successful is to enumerate all possible applications of the DPO rule to the reactant graph *G* and to check whether there is at least one case in which the expected products are generated. We use MØD for this purpose. This tool implements a complete implementation of DPO graph rewriting and can enumerate all matches. Residual ambiguities may result in more than one non-isomorphic solution.

### Completion of the atom-to-atom map (AAM)

Since map $\alpha :V(L)\rightarrow V(R)$ contains in particular all atoms with bonds that change, it is straightforward to extend $\alpha$ to an atom-to-atom map for the full reactant and product graphs. The key observation is that for every atom in $V(G)\setminus V(L)$ neither the adjacencies nor the edge labels change. Thus, a variant of VF2 can be used that can be initialized with $\alpha$, i.e., by *declaring* $M=\{(x,\alpha (x))| x\in V(L)$ a consistent set of matches. A vertex match $xy\notin M$ can then be appended to *M* if and only if edges and edge labels are preserved for $xx'$ and $y\alpha (x')$ for all $(x',\alpha (x'))\in M$. Using the assumption that all vertices with changes in their neighborhoods are already included in the initial set *M*, it follows that there is an extension of the matching for which every vertex in *V*(*G*) and every vertex in *V*(*H*) is eventually matched. VF2 thus terminates as if it had found an isomorphism. In case previous sets have led to multiple possible results for $\alpha$, it is possible to use the extensibility described here as a filter criterion. More precisely, a set of matches *M* that is not extended to a bijection by VF2 cannot correspond to a correct DPO rule for the reactions.

Completion of AAMs comprises two separate steps. First, a DPO rule is applied to all reactants, resulting in hypergraphs with hyperedges that represent possible reactions resulting from the DPO rule. Here, we retain only the hyperedges that connect the reactants to products of a known reaction. For these, an AAM is defined directly by the application of the DPO rule. This approach necessarily fails if the DPO rule does not cover the complete reaction center.

In the second step, we therefore use VF2 to enumerate all subgraph isomorphisms of the educt graph of the reaction center within the educt side to the reaction graph. The same procedure is performed for the product side. We then generate all combinations of isomorphisms of the educt and product side to obtain the list of all possible rule applications. Each of these represents a partial atom map limited to the atoms of the reaction center. An Integer Linear Program described in detail in [31] is then used to minimize the number of remaining bonds that change. This method yields a complete AAM for the full reaction.

### Implementation

The workflow for translating the RCLASS specification into a DPO rule is implemented by the Python program laveau.^2^ It uses a reaction, its structural formulas, and the RCLASS for each reactant/product pair and input. The program attempts to construct the corresponding DPO rule using the algorithms. The laveau toolkit consists of five scripts that can be run consecutively. In the first step, the RDM codes are transformed into their corresponding graph representations. The second script assembles the graphs into RDM graphs. These are assembled further into pairwise RDM pattern graphs by the third script. In script four, all pairwise RDM pattern graphs are then mapped to the corresponding reactant sets. The result is a graph representation of the reaction rule that is translated into the explicit DPO rule format. In the fifth and final step, the AAMs are produced by application of the rule on the reactants. The final step requires the MØD package (https://jakobandersen.github.io/mod/). The number of combinations in step four can increase very rapidly, which is why a filter function has been included that skips reactions with too many combinations (default is 1000 graphs per reaction side). The same issue also affects the application of the DPO rules with MØD. Here, a time limit of two hours has been set as the default. If the generation of all products by the rule exceeds this time limit, the process is aborted, and the next rule is started. A more detailed overview of all combinations and the filters used can be found in the Appendix.

## Results

### Transformation of the KEGG RCLASS collection

The current version of the KEGG reactions database (Last Release 109.1, March 1, 2024) contains 3195 RCLASSes collectively describing 12059 reactions. A total of 2960 RCLASSes (92.6%) could be converted from their RDM code into *RDM pattern graphs*. The remaining 235 rules contain type graphs with atoms labeled as ’undefined atoms’. Since they do not define local structures, it is not feasible to include them. Almost a fifth of the tractable cases (588 of 2960) yield more than one *RDM pattern graph*. Both unique and ambiguous cases were tested for embedding into the molecular graphs. A *pairwise pattern graph* could be computed for 2233 of the 2960 *RDM pattern graphs*. In 644 cases, i.e., about 29% of the tractable data, the result was ambiguous in the sense that more than one rule is consistent with the information of the RDM codes.

Of all reactions listed in KEGG, 1495 were not linked to any RCLASS and thus could not be processed. Furthermore, 1102 reactions link to RCLASSes with undefined atoms. These RCLASSes are not suitable for our purpose and were not considered for the extraction of reaction rules. In 1849 cases, at least one of the linked RCLASSes could not be constructed in the previous step due to reasons stated above. In addition, 2880 reactions had to be filtered out because an exhaustive enumeration of all combinations of matching patterns was not feasible. 57 reactions contain molecules without any structural information in reaction centers. Therefore, they could also not be processed further. Out of the 4676 remaining reactions, we were able to generate DPO rules for 3612.

*laveau* first generates all possible DPO rules that satisfy the conditions specified in the Methods section. Depending on the component structures. This can result in several DPO rules, of which some may not represent valid AAMs; these are removed in a filtering step. For 12 reactions, we found multiple solutions. In five of these cases, symmetric molecules were the source of ambiguity, while tautomerism was observed as the cause in six cases. In one single reaction (R03930), two different DPO rules could be validated that represent two different reaction mechanisms. These variants were also marked in the lists of AAMs with ’S’ for symmetries and ’T’ for tautomerism. In addition, for 77 reactions, several AAMs could also be generated and thus validate the rules, but these reactions differed only in the number of hydrogen exchanges. Here, the rules were filtered to the minimum number of hydrogen exchanges.

### Atom-to-atom maps of KEGG reactions

Overall, we obtained 1594 global atom-to-atom maps. For these reactions, all products could be generated by the DPO rule, and the AAM covers the entire reaction. In addition, we obtained 253 partial atom-to-atom maps for which the AAM only covers a subset of the molecules taking part in the reaction. We classified partial AAM depending on whether missing molecules or partial molecules are co factors, single protons, single Phosphates, abbreviated functional groups, or part of the reaction mechanism. Partial AAMs that only cover co factors were removed from the data set. For 2380 reactions, it was not possible to derive correct AAMs from any of the DPO rule variants. As described above, an alternative tool was used to try to complete DPO rules and thus obtain an AAM. This was successful for 312 reactions. However, only 29 of these had a unique AAM, while the inferred AAM remains ambiguous for the overwhelming majority of these cases.

In the second attempt to complete AAMs, the DPO rule is matched to the reactants and products at each suitable position, and the rest of the AAM is filled in by isomorphy tests. This allows a wide variety of embeddings of the rules, in particular for small reaction centers and molecules with repeating atomic sequences, which then leads to a high number of permissible maps. In 92% of the evaluated rules, multiple maps could be generated. Specifically, more than 10 maps were produced in 59% of cases, over 100 in 27%, and in 2% of the cases, the number of distinct maps exceeded 1000.

As no gold standard dataset is available, the correctness of DPO rules cannot be conclusively verified. However, consistency with previously cataloged mechanisms can be inspected. By design, laveau produced only DPO rules congruent to and within the margin of error inherent to KEGG’s annotation. The completion step within laveaus workflow guarantees consistency of extracted DPO rules to associated reactions in KEGG. To further validate the results, the global AAMs were compared with the widely used RXNMapper [3], revealing that certain enzymatic reaction mechanisms cannot be captured by machine learning methods such as RXNMapper, but can be represented by laveau. The detailed results of the analysis can be found in the Appendix.

These results demonstrate the effectiveness of DPO rules in generating comprehensive atom-to-atom maps, but they also reveal inherent limitations, especially when dealing with highly complex and large reactions. Challenges such as residual labeling, intricate co-factor involvement, and ambiguous embeddings in reactions with extensive molecular structures highlight the need for advanced mapping strategies to ensure complete and accurate coverage.

### Limitations of laveau

**Fig. 10:**
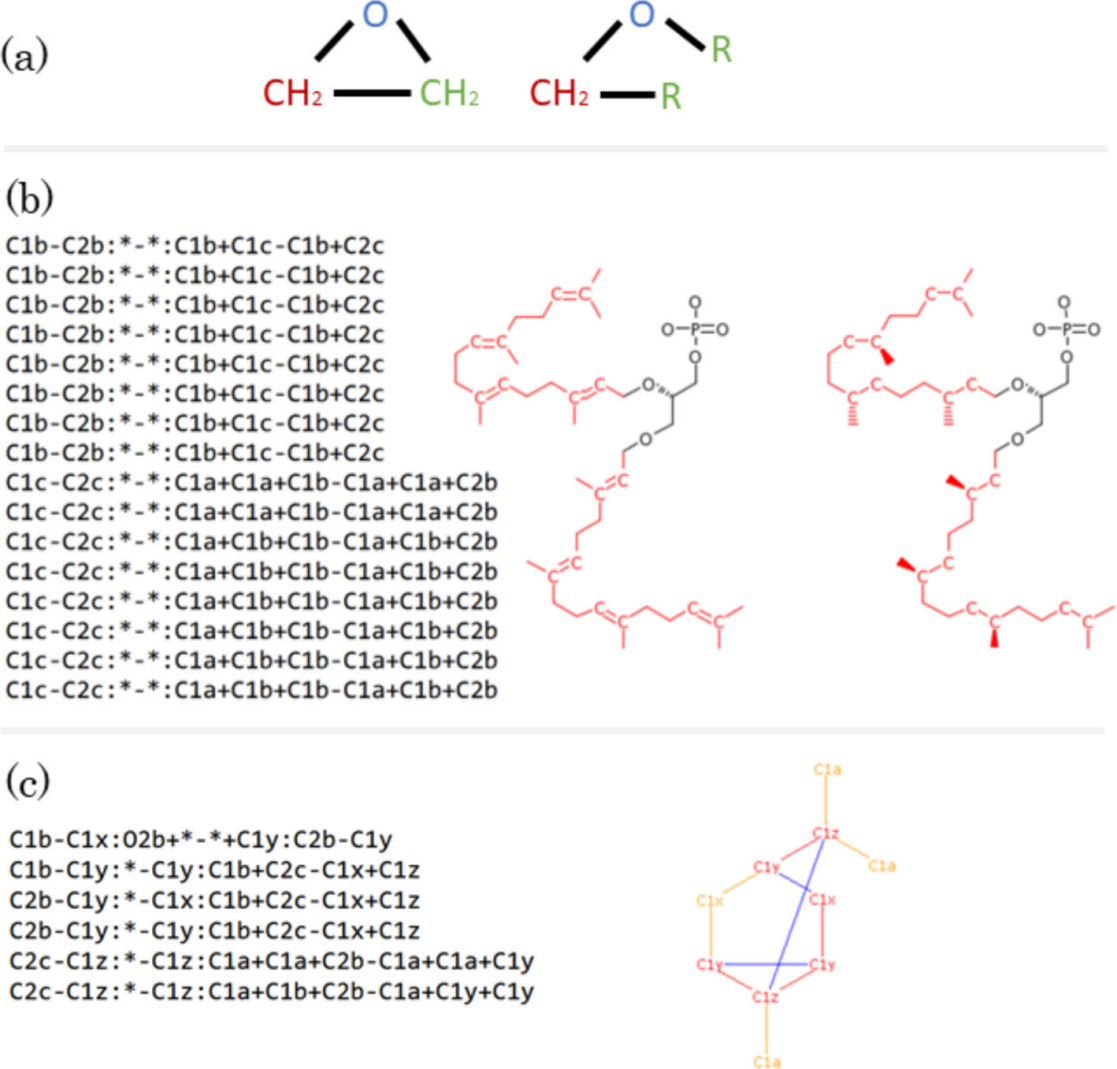
Examples of RCLASSes that cannot be transformed. **a** The embedding fails in molecules with 3-rings. These are not directly encoded in the RDM data and appear as paths. On the left-side is the molecule, and on the right side is one of three right sub-graphs of RCLASS RC01334. The colors indicate the matching of the atoms. Both wildcard atoms would need to match the same carbon atom. The embedding thus would not be a subgraph monomorphism. **b** RCLASS RC03134 consists of 16 R-atoms, which, however, only differ in two different environments. Such polymer structures lead to an exploding number of combinations that can no longer be processed in a practical way. **c** In the RCLASS RC02760, the variance of the R-atom environment is stronger, but the strong branching leads to a very high number of different variants of the mapping, which causes the transformation of the single RCLASS to take several days to process

The analysis of reaction mechanisms using laveau is subject to some limitations, particularly when handling complex reaction patterns and unbalanced reactions in the KEGG database. In 727 cases, it was not possible to embed an *RDM pattern graph* into any molecular graph. This limitation is especially apparent in reactions involving the formation or breaking of three-ring structures, such as epoxides. The primary cause is that RDM data does not account for instances where two residues overlap (see Fig. 10a).

Another challenge is that RCLASS entries with numerous similar environments around multiple R-atoms (Fig. 10b) or with highly branched R-atoms (Fig. 10c) can result in a combinatorial explosion of possible embeddings within the molecular graphs. In such cases, the computation becomes infeasible or prohibitively costly. Overall, 164 RDM codes with over 1000 distinct variations of *RDM pattern graphs* embeddings were identified.

Another limitation in generating DPO (Double Push Out) rules is posed by unbalanced reactions found in the KEGG database, where entire reactants are missing. For instance, in reaction R07733, ononin is converted to formononetin, yet the glucose that is removed in this process is absent from the product side. Altogether, 1064 reactions were identified that could not be converted into meaningful DPO rules for this reason. In some instances, it is also impossible to generate molecular graphs because of missing structural information, e.g., for chemically modified proteins such as [SoxY protein]-S-sulfanyl-L-cysteine (C21898). No additional efforts were made to resolve such cases, as they are overall rare.

**Fig. 11:**
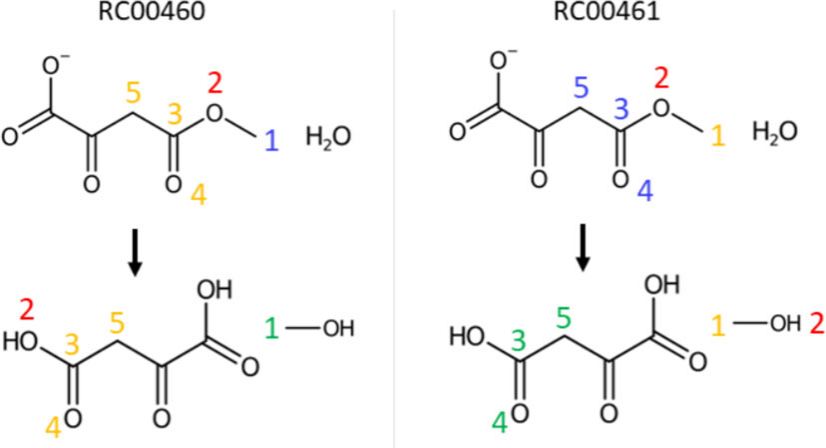
Reaction R01144 is an example with conflicting RDM codes in its atom-to-atom maps. The numbers indicate the partial atom-to-atom map from the corresponding RDM codes. Red numbers represent R-atoms, yellow M-atoms, blue D-atoms, and green corresponding D-atoms found by laveau. Merging the partial atom-to-atom maps from the two linked RDM codes is not possible here because the O2 atom is mapped in different ways

Moreover, cases with more than one RCLASS linked to a single reaction sometimes produced conflicting atom-to-atom mappings due to differing RDM codes representing separate mechanisms (see Fig. 11). No further attempts were made to determine if one of these mechanisms was correct or if there might be enzymes that naturally produce alternative atom-to-atom mappings.

## Concluding remarks

The reconstruction of atom-to-atom maps and reaction rules data from the information in the KEGG RCLASS data turns out to be a difficult graph reconstruction problem, which is aggravated by the fact that some of the elementary building blocks are not uniquely defined. We developed an exact algorithm that first converts the RDM codes into (sets of) small graphs and then determines the possible embeddings of these graphs in the reactant and product molecules. Compatible embeddings determine a subgraph. Our approach, which is implemented in python tool laveau, proceeds by exhaustive enumeration. It succeeds in about three-quarters of the cases. Exceptions are cases with ’undefined’ atoms, epoxids, and similar cases with three rings, and RCLASSes with a very large number of R-atoms. The latter corresponds to a complex multi-step reaction with large net changes between reactants and products.

In a non-negligible fraction of the cases, we obtain multiple solutions. The encoding of reaction mechanisms in terms of RDM codes is thus a lossy encoding that cannot be fully inverted. The ability to retrieve atom-to-atom mapping data, particularly in biochemical systems, however, is important for at least two reasons.

First, generic atom-to-atom mapping tools tend to perform much less well on metabolic reactions than on reactions from general organic synthesis data. A likely reason for this observation is transient bonds formed with the enzyme. The actual mechanism of the reaction, therefore, is not completely described by reactants and products. Methods trained on or designed for reactions without catalysts thus perform suboptimally.

Second, atom-to-atom maps are of particular relevance in metabolic systems because they form the starting point for constructing atom transition networks [35]. These play a key role, e.g., in isotope tracing experiments. From this point of view, the most valuable result of this contribution is the DPO rules and atom-to-atom maps that were constructed by laveau. Since new RCLASS data virtually require the preparation of an expert-curated atom-to-atom map for the reaction center, we would hope that much more direct information would (also) become available.

The current implementation of laveau handles a large fraction of the RCLASS data but does not achieve complete coverage. Undetected changes in the number of binding hydrogens or changes in the bond types of atoms adjacent to a D-atom may lead to DPO rules that do not completely explain reactant/product pairs. Such rules could potentially be corrected by more sophisticated methods able to cover such incomplete reaction centers. Moreover, algorithmic improvements appear to be possible that speed up the processing of RCLASSes with many R-atoms. However, the number of RCLASS data records does not seem to grow rapidly; hence, computational efficiency is of practical relevance only for the rather limited number of RCLASSes where the computation is infeasible. For the few records containing compounds that cannot be converted to graphs automatically, as well as some other rare exceptions, a manual reconstruction of the atom-to-atom is probably less effort than attempting to extend laveau so that it handles all RCLASS entries.

An interesting question arising from the problem of reconstructing atom-to-atom maps from RDM codes is whether there exists an alternative representation of the relevant subgraph $\Psi$ of the ITS of a reaction.

More precisely, we ask whether there exists a (multi)set $\mathcal {R}$ of subgraphs of $\Psi$ such that (1) $\Psi$ can be (unambiguously) reconstructed from $\mathcal {R}$, (2) the subgraphs in $\mathcal {R}$ are “local” in $\Psi$ (e.g., in the sense of having a fixed small diameter). In general, such *shotgun graph assembly* problems do not have a unique solution [26]. It remains an open question, however, to what extent the moderate sizes of chemically relevant reaction centers $\Psi$ and the additional vertex end edge labels might reduce ambiguities of such sets of small covering subgraphs.

## Additional file

Supplementary file 1 (pdf 86 KB)

Supplementary file 2 (pdf 63 KB)

Supplementary file 3 (png 18 KB)

Supplementary file 4 (pdf 62 KB)

Supplementary file 5 (png 17 KB)

Supplementary file 6 (pdf 731 KB)

## Figures and Tables

**Fig. 12:**
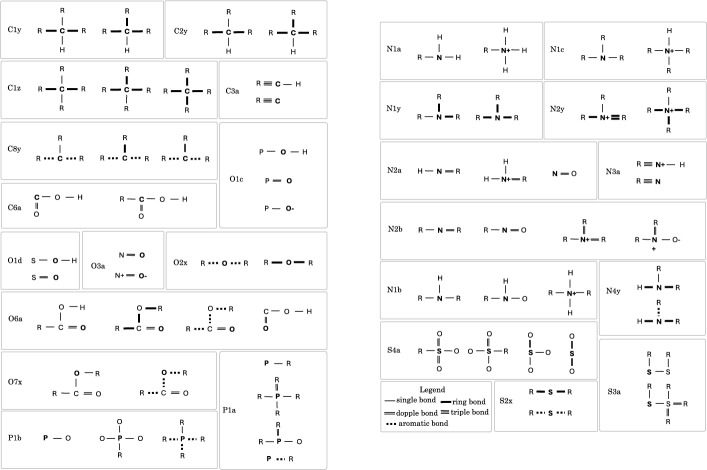
Alternative patterns for carbons, oxygens and, phosphorus, nitrogens, and sulphur. Bold highlighted atoms indicate the R-atom. Each box shows the patterns developed by [7, 8] as well as the ones we created for each label

**Fig. 13:**
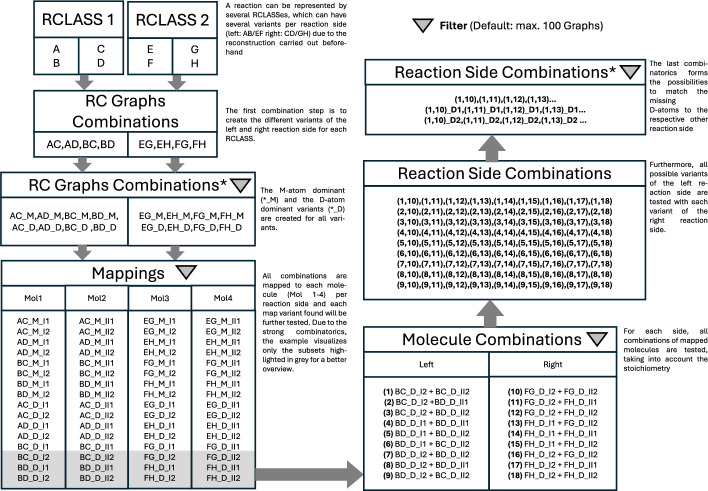
Overview of all combinatorial steps in the reconstruction of reaction rules. To illustrate the combinatorial behavior with an example, it was determined that there are always two instances defined for each variation. Of course, these differ from case to case in reality

**Fig. 14:**
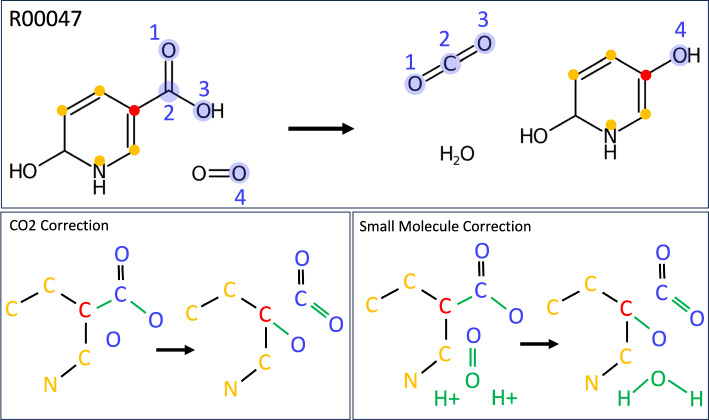
Illustration of the correction of the DPO rules for reactions with small molecules. The corrections are marked in green. R-atoms red, D-atoms blue, and M-atoms yellow. An example reaction is shown (top), which must be corrected. The D-atoms with the IDs 1–3 are not found by the algorithm because they contain a change from a single bond to a double bond. The first correction recognizes this special case so that the DPO rule recognizes and corrects it (bottom left). The second correction adds the missing parts of the mechanism (bottom right)

**Fig. 15:**
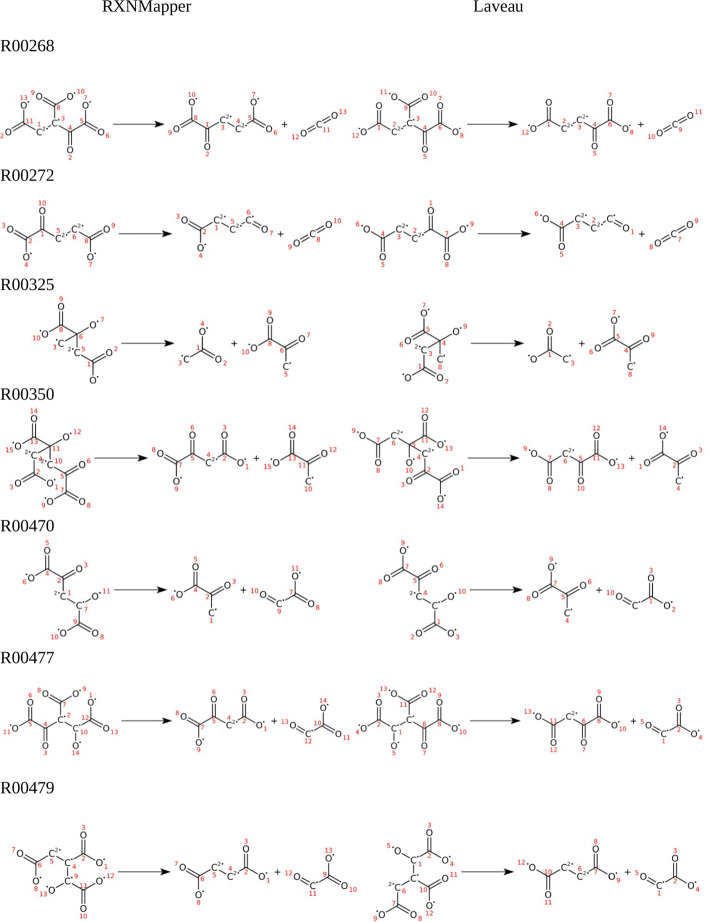
Visualization of all reactions where laveau (left) and RXNMapper (right) produced different AAMs in the comparative study. Manual inspection suggests that laveau produced the more plausible mechanism for all reactions

## Data Availability

The software and data are publicly available at the following Github repository: https://github.com/NoraBeier/laveau.

## Appendix

### Inconsistencies in RDM pattern

In some cases, RDM codes could not be unpacked since bond types could not be matched between adjacent type graphs. In order to handle such cases, we defined alternative patterns as shown in Fig. 12 for some of the graph types. The laveau software attempts to use these if the unpacking of RDM codes failed with the standard interpretation of labels as defined in [7, 8]. The reason for this modification is that the existing graph types do not represent all possible molecular configurations. For example, protonation variants of a molecule, e.g., in sulfates, are typically represented by a single type graph (S4a), but they appear in multiple protonation forms in the reaction data. In the case of sulphates, for instance, we find $\text {O}-\text {S}(-\text {O})\text {=O}$ and $\text {O}-\text {S(=O)=O}$. Nevertheless, the molecules that contain them still retain their molecular identity. Therefore, three additional *type graphs* had to be formulated to account for these variants. Another significant reason is the differentiation between ring bonds and aromatic bonds. During the development of laveau, it became evident that many *type graphs* representing ring bonds also depicted aromatic bonds in certain cases (e.g., O2x). In some instances, even a single bond had to be redefined as either a ring bond or aromatic bond, as in the case of O61, to accurately represent all different reaction centers for which this code is used. This approach ensures a more comprehensive representation of molecular structures, enabling a more accurate unpacking and interpretation of RDM codes.

### Combinatorial explosion and filter for reconstruction of reaction rules

During the development of laveau it turned out that the reconstruction of reaction rules sometimes leads to a combinatorial explosion, i.e., the number of possible alternatives that have to be tested exceeds the available computer memory. Fig. 13 shows the individual combination steps again and the filters (marked as triangles) that are applied by default for 100 graphs.

### Correction of DPO rules for reactions with small molecules

In order to create functional reaction rules even with small molecules, two additional functions have been added to laveau. The first is concerned with the special case “elimination/addition of CO2”. Without this function, there is a mismatch of the D-atom sub-graphs, as the cleavage of carboxyl groups to CO2 results in a bond change. More precisely, between the carbon and the single-bonded oxygen to a double bond. The function, therefore, checks after all carboxyl group D-atom sub-graph mismatches whether a CO2 molecule is present on the other side of the reaction. If this is the case, the D-atom sub-graph is then mapped to the CO2 molecule. The second function adds important reaction-mechanism content in cases where D-atom sub-graphs consist of a single atom. Here, the neighbors of the D-atom sub-graph and their bonds are considered. For example, in Fig. 14, the O$^4$ atom of the product originates from the O2 molecule, where the oxygen D-atom has a neighbor with a double bond. This additional neighbor, including the bond on the corresponding side, is also included in the DPO rule. Since this is a D-atom sub-graph with only one node, the bond to its neighbors must be released in the reaction. Since no other RDM code indicates that this neighbor forms new bonds with other atoms, the free bonds connect to hydrogens.

### Comparative analysis of AAM predictions with RXNMapper

Global AAMs generated by laveau were compared to those produced by RXNMapper [3] for the same reaction. For this purpose, SMILES representations without atom mapping were extracted for each AAM and subsequently used as an agnostic input for RXNMapper. As hydrogen mappings are related only to adjacent non-hydrogen atoms and can differ mainly by chemically insignificant rotations in representation, hydrogen atoms were removed from the mappings of both tools. In addition, reactions for which RXNMapper produced only incomplete mappings were excluded from the comparison. This procedure yielded a total of 125 reactions, which were then compared using EEquAAM [36]. As a result, 25 AAMs of laveau were identified that differ from those produced by RXNMapper (Fig. 15). By manual inspection, we found that in all cases, RXNMapper produced an implausible reaction mechanism, while laveaus AAM appears sound.

## Footnotes

1 see https://jakobandersen.github.io/mod/graphModel/index.html.

2 Marie Laveau (1801–1881), Voodoo queen of New Orleans.

## References

[1] Chen WL, Chen DZ, Taylor KT. Automatic reaction mapping and reaction center detection. WIRES Comp Mol Sci. 2013;3:560–93.

[2] Jaworski W, Szymkuć S, Mikulak-Klucznik B, Piecuch K, Klucznik T, Kaźmierowski M, et al. Automatic mapping of atoms across both simple and complex chemical reactions. Nat Comm. 2019;10:1434. 10.1038/s41467-019-09440-2 PMC6441094 30926819

[3] Schwaller P, Hoover B, Reymond JL, Strobelt H, Laino T. Extraction of organic chemistry grammar from unsupervised learning of chemical reactions. Sci Adv. 2021;7:eabe4166. 33827815 10.1126/sciadv.abe4166 PMC8026122

[4] Starke C, Wegner A. MetAMDB: metabolic atom mapping database. Metabolites. 2022;12:122. 35208198 10.3390/metabo12020122 PMC8878866

[5] Phan TL, Weinbauer K, Laffitte MEG, Pan Y, Merkle D, Andersen JL, et al. Syntemp: efficient extraction of graph-based reaction rules from large-scale reaction databases. J Chem Inf Model. 2025;65(6):2882–96. 40019281 10.1021/acs.jcim.4c01795 PMC11938280

[6] Kanehisa M, Goto S, Sato Y, Kawashima M, Furumichi M, Tanabe M. Data, information, knowledge and principle: back to metabolism in KEGG. Nucleic Acids Res. 2014;42:D199-205. 24214961 10.1093/nar/gkt1076 PMC3965122

[7] Kotera M, Okuno Y, Hattori M, Goto S, Kanehisa M. Computational assignment of the EC numbers for genomic-scale analysis of enzymatic reactions. J Am Chem Soc. 2004;126:16487–98. 15600352 10.1021/ja0466457

[8] Hattori M, Okuno Y, Goto S, Kanehisa M. Development of a chemical structure comparison method for integrated analysis of chemical and genomic information in the metabolic pathways. J Am Chem Soc. 2003;125(39):11853–65. 14505407 10.1021/ja036030u

[9] Shimizu Y, Hattori M, Goto S, Kanehisa M. Generalized reaction patterns for prediction of unknown enzymatic reactions. Genome Inform. 2008;20:149–58. 19425130

[10] Yamanishi Y, Hattori M, Kotera M, Goto S, Kanehisa M. E-zyme: predicting potential EC numbers from the chemical transformation pattern of substrate-product pairs. Bioinformatics. 2009;25(12):i179-86. 19477985 10.1093/bioinformatics/btp223 PMC2687977

[11] Ehrig H, Ehrig K, Prange U, Taenthzer G. Fundamentals of Algebraic Graph Transformation. Berlin: Springer-Verlag; 2006.

[12] Andersen JL, Flamm C, Merkle D, Stadler PF. Inferring Chemical Reaction Patterns Using Graph Grammar Rule Composition. J Syst Chem. 2013;4:4.

[13] Andersen JL, Flamm C, Merkle D, Stadler PF. A Software Package for Chemically Inspired Graph Transformation. In: Echahed R, Minas M, editors. Graph Transformation, ICGT 2016. vol. 9761 of Lecture Notes Comp. Sci. Berlin, Heidelberg, D: Springer Verlag; 2016. p. 73-88.

[14] Andersen JL, Flamm C, Merkle D, Stadler PF. Chemical transformation motifs–modelling pathways as integer hyperflows. IEEE/ACM Trans Comp Biol. 2019;16:510–23. 10.1109/TCBB.2017.2781724 29990045

[15] Fujita S. Description of organic reactions based on imaginary transition structures. 1. Introduction of new concepts. J Chem Inf Comput Sci. 1986;26:205–12.

[16] Körner R, Apostolakis J. Automatic determination of reaction mappings and reaction center information. 1. The imaginary transition state energy approach. J Chem Inf Model. 2008;48:1181–9. 18533713 10.1021/ci7004324

[17] Hoonakker F, Lachiche N, Varnek A, Wagner A. A representation to apply usual data mining techniques to chemical reactions-illustration on the rate constant of reactions in water. Int J Artif Intelligence Tools. 2011;20:253–70.

[18] Nugmanov RI, Mukhametgaleev RN, Akhmetshin T, Gimadiev TR, Afonina VA, Madzhidov TI, et al. CGRtools: python library for molecule, reaction, and condensed graph of reaction processing. J Chem Inf Model. 2019;59(6):2516–21. 31063394 10.1021/acs.jcim.9b00102

[19] Kelly PJ. A congruence theorem for trees. Pacific J Math. 1957;7:961–8.

[20] Harary F. On the reconstruction of a graph from a collection of subgraphs. In: Fiedler M, editor. Theory of Graphs and its Applications (Proc. Sympos. Smolenice, 1963). Praha: Československá akademie věd.; 1964. p. 47-52.

[21] O’Neil PV. Ulam’s conjecture and graph reconstructions. Amer Math Monthly. 1970;77:35–43.

[22] Bondy JA, Hemminger RL. Graph reconstruction—a survey. J Graph Th. 1977;1:227–68.

[23] Kostochka AV, West DB. On reconstruction of graphs from the multiset of subgraphs obtained by deleting vertices. IEEE Trans Inf Th. 2021;67:3278–86.

[24] McKay B. Reconstruction of small graphs and digraphs. Austras J Combin. 2022;83:448–57.

[25] Bollobás B. Almost every graph has reconstruction number three. J Graph Theory. 1990;14:1–4.

[26] Mossel E, Ross N. Shotgun assembly of labeled graphs. IEEE Trans Network Sci Eng. 2019;6(2):145–57.

[27] Sedláček J. The reconstruction of a connected graph from its spanning trees. Math Časopis. 1974;24:307–14.

[28] Lack S, Sobociński P. Adhesive and quasiadhesive categories. RAIRO-Theor Inform Appl. 2005;39(3):511–45.

[29] Behr N, Sobociński P. Rule Algebras for Adhesive Categories. In: Ghica D, Jung A, editors. 27th EACSL Annual Conference on Computer Science Logic (CSL 2018). vol. 119 of Leibniz International Proceedings in Informatics (LIPIcs). Schloss Dagstuhl–Leibniz-Zentrum fuer Informatik; 2018. p. 11.

[30] Behr N, Krivine J. Compositionality of rewriting rules with conditions. Compositionality. 2021;3:2.

[31] González Laffitte ME, Weibauer K, Phan TL, Beier N, Domschke N, Flamm C, et al. Partial its graphs: a formal framework for research and analysis of atom-to-atom-maps of unbalanced chemical reactions and their completions. Symmetry. 2024;16(9):1217.

[32] Laffitte MEG, Phan TL, F SP. Extension of Partial Atom-to-Atom Maps: Uniqueness and Algorithms. In: Brejová B, Patro R, editors. In 25th International Conference on Algorithms for Bioinformatics (WABI 2025). vol. 344 of Leibniz International Proceedings in Informatics (LIPIcs). Dagstuhl, Germany: Schloss Dagstuhl–Leibniz-Zentrum fuer Informatik; 2025. p. 12.

[33] Fooshee D, Andronico A, Baldi P. ReactionMap: an Efficient Atom-Mapping Algorithm for Chemical Reactions. J Chem Inf Model. 2013;53:2812–9. 24160861 10.1021/ci400326p

[34] Heinonen M, Lappalainen S, Mielikäinen T, Rousu J. Computing atom mappings for biochemical reactions without subgraph isomorphism. J Comput Biol. 2011;18:43–58. 21210731 10.1089/cmb.2009.0216

[35] Haraldsdóttir HS, Fleming RMT. Identification of conserved moieties in metabolic networks by graph theoretical analysis of atom transition networks. PLoS Comput Biol. 2016;12:e1004999. 27870845 10.1371/journal.pcbi.1004999 PMC5117560

[36] González Laffitte ME, Beier N, Domschke N, Stadler PF. Comparison of atom maps. MATCH Comm Math Comp Chem. 2023;90:75–102.

