# Supplementary figures and images for: Computing double-pushout graph transformation rules and atom-to-atom maps from KEGG RCLASS data
Source: Algorithms Mol Biol. 2026 Jan 29;21:3. doi: 10.1186/s13015-025-00294-6 (PMC12949509; doi:10.1186/s13015-025-00294-6)

R00047

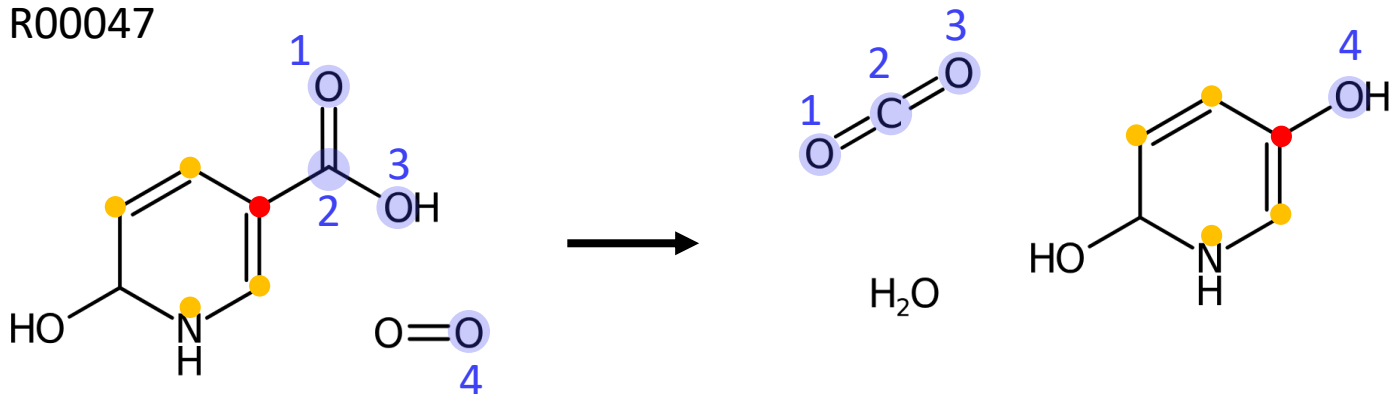

CO2 Correction

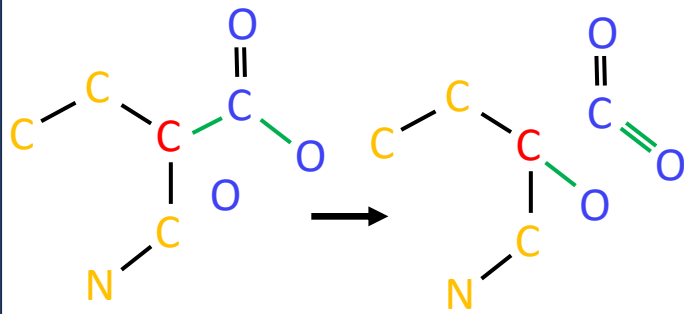

Small Molecule Correction

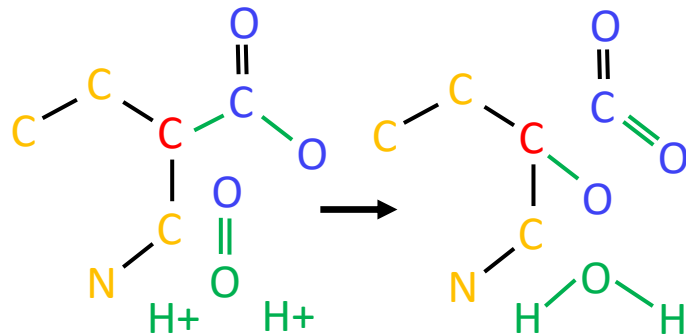

Supplement: Supplementary file 1 — (pdf 86 KB) [file 13015_2025_294_MOESM1_ESM.pdf]

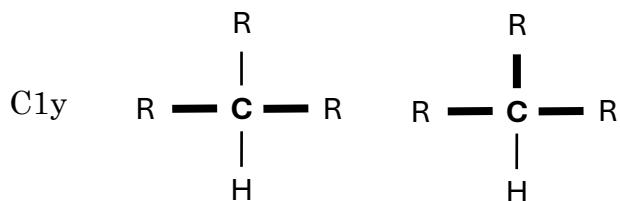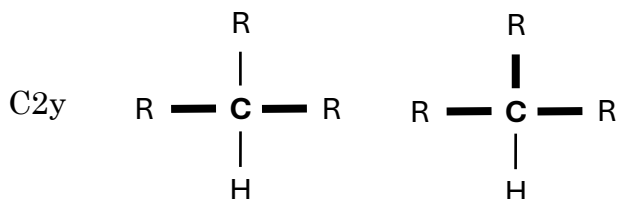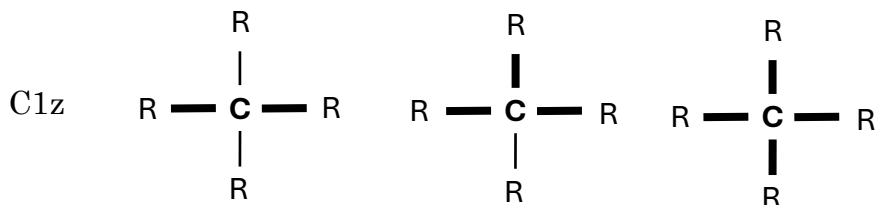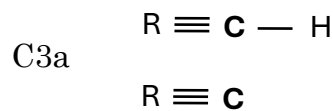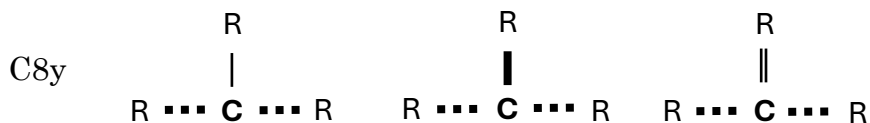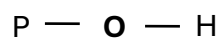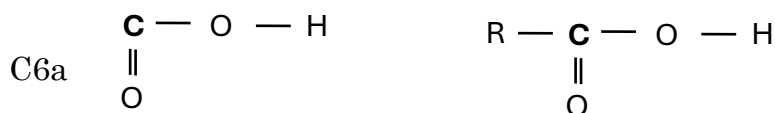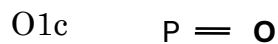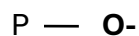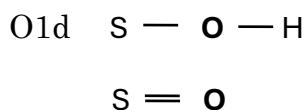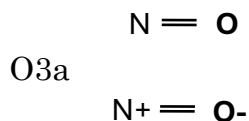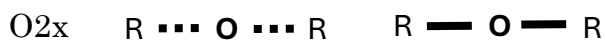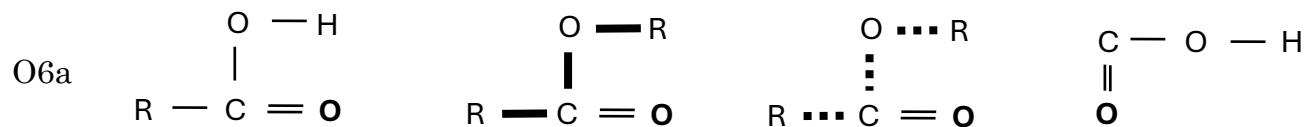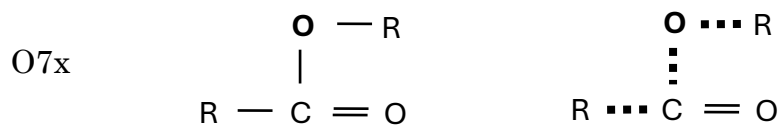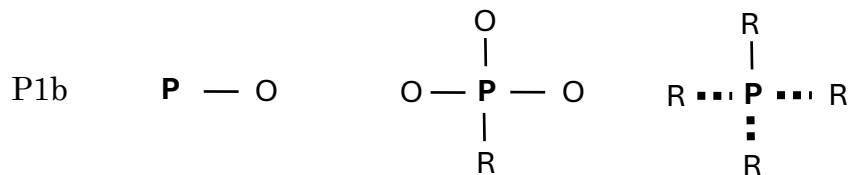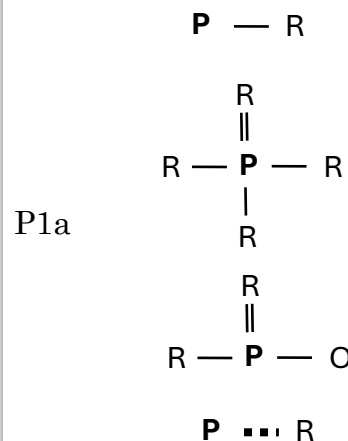

Supplement: Supplementary file 2 — (pdf 63 KB) [file 13015_2025_294_MOESM2_ESM.pdf]

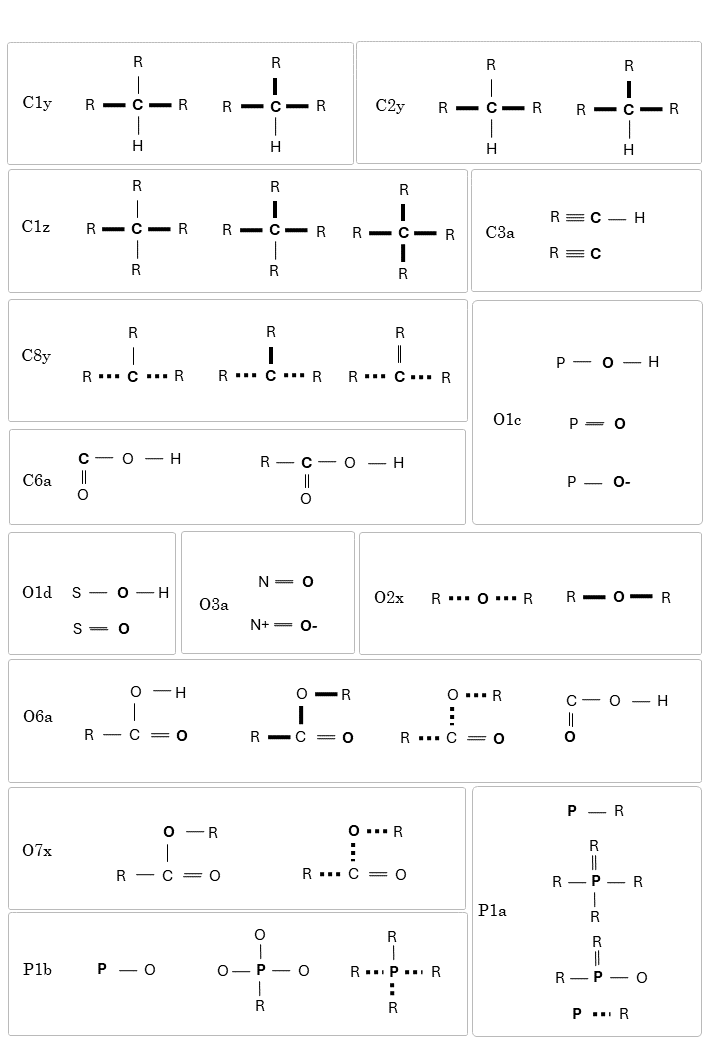

Supplement: Supplementary file 3 — (png 18 KB) [file 13015_2025_294_MOESM3_ESM.png]

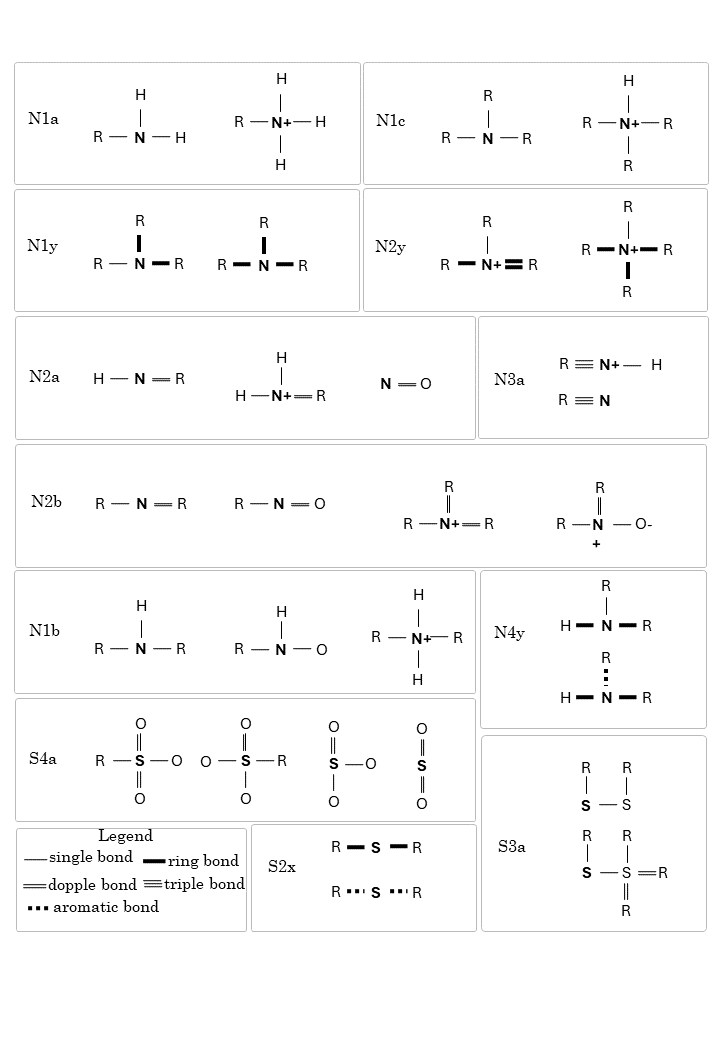

Supplement: Supplementary file 5 — (png 17 KB) [file 13015_2025_294_MOESM5_ESM.png]

RXNMapper

Laveau

R00268

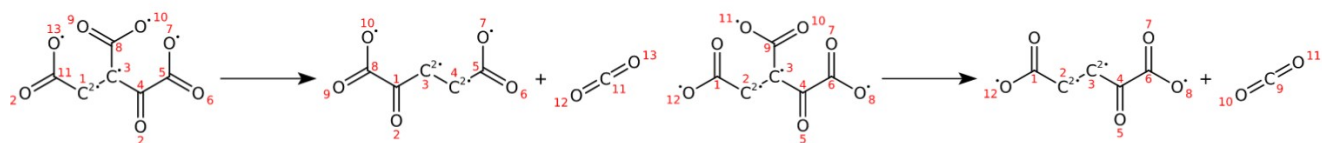

R00272

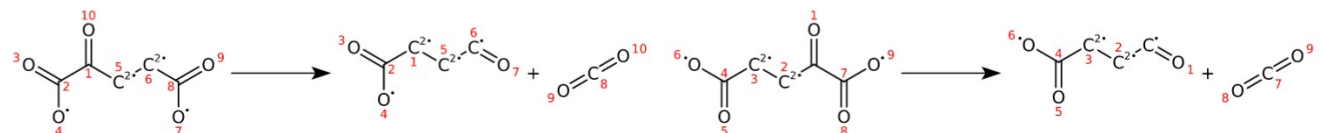

R00325

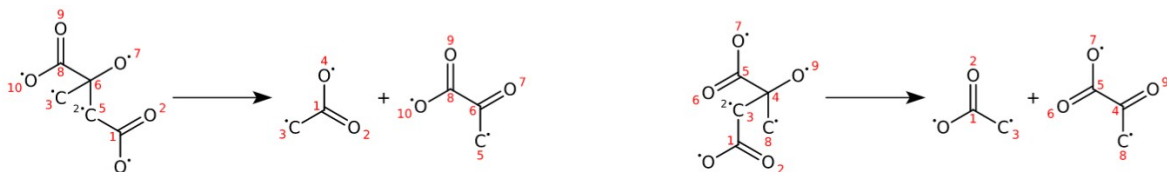

R00350

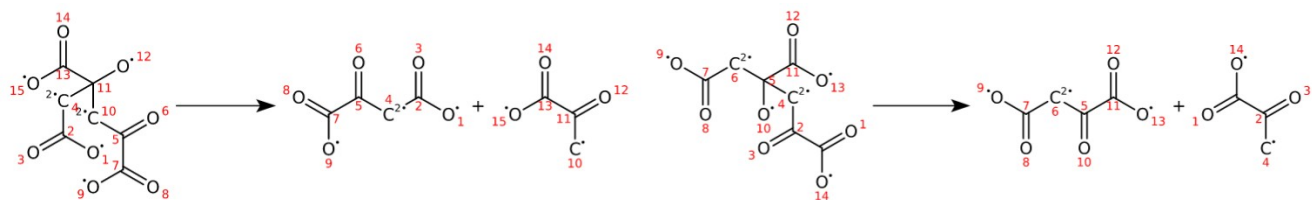

R00470

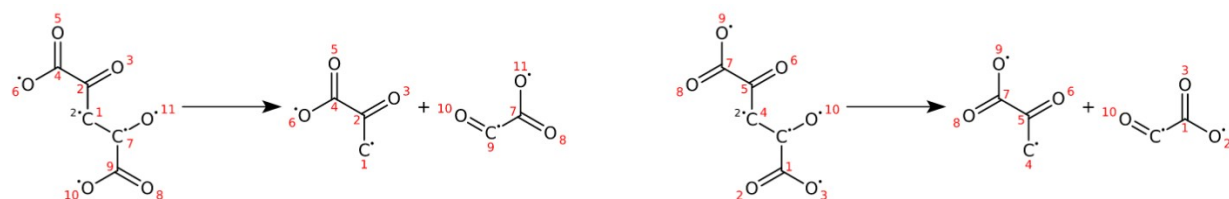

R00477

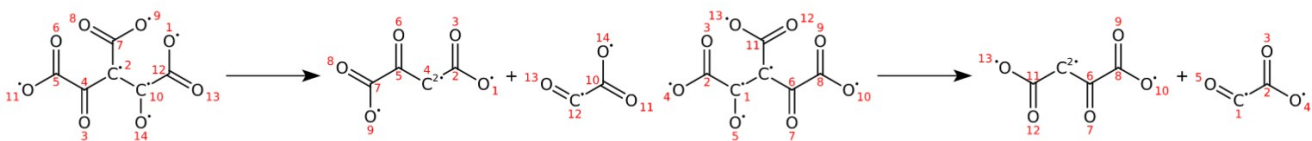

R00479

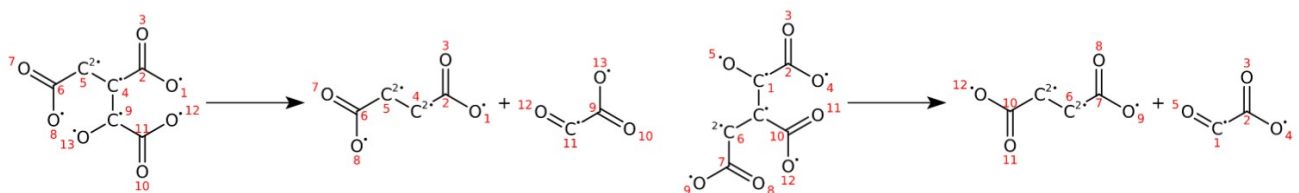

R01645

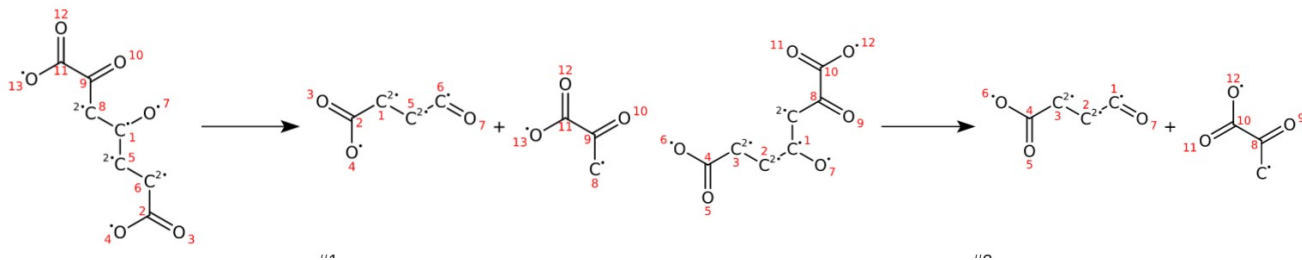

R02009

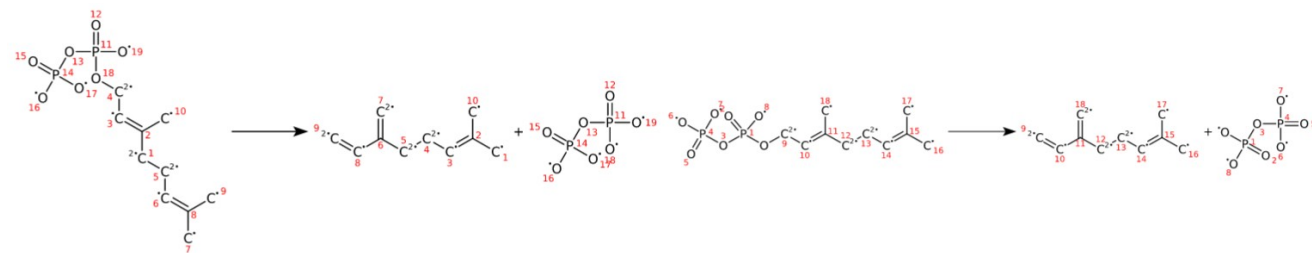

R02937

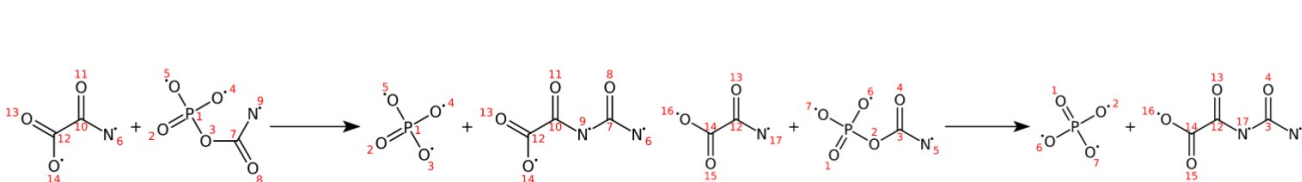

R03070

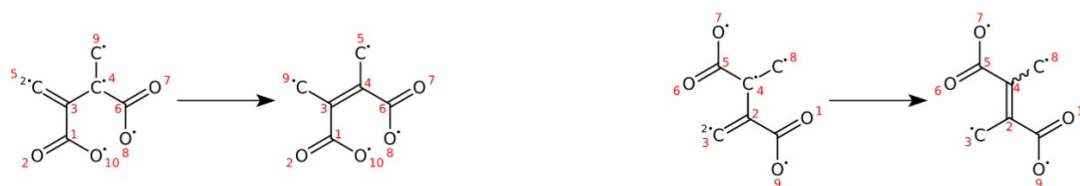

R03127

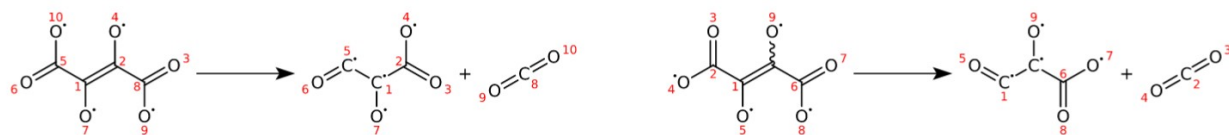

R03747

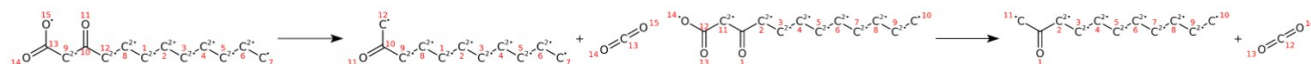

R04134

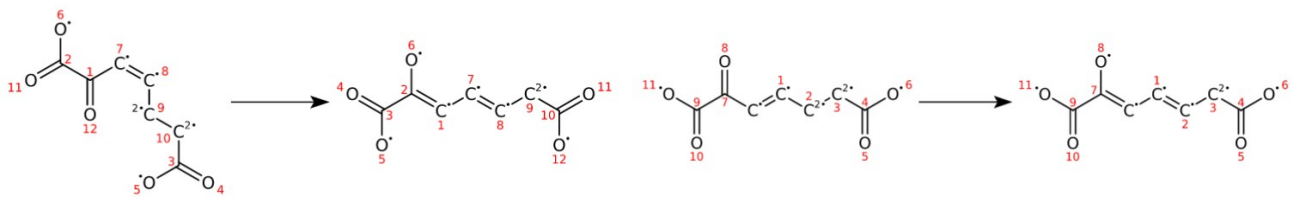

R04380

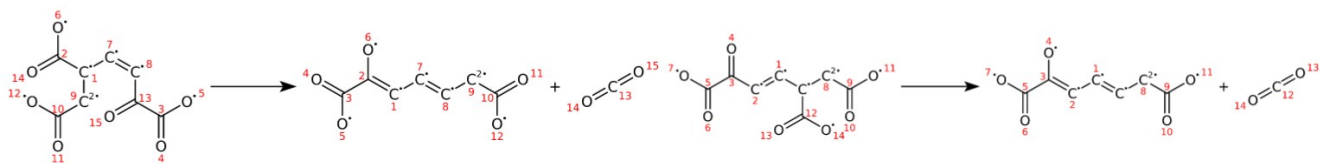

R04482

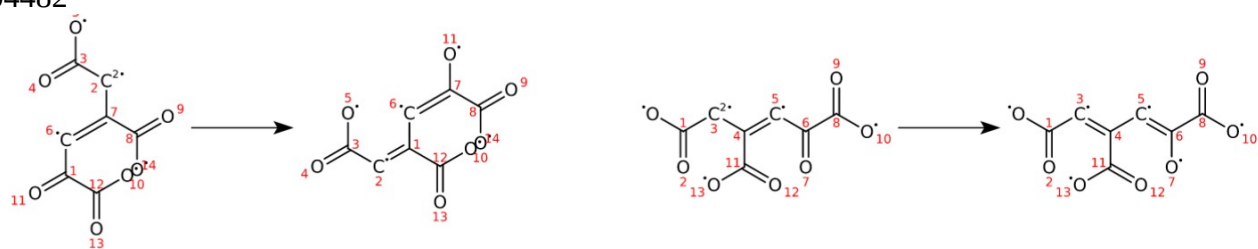

R04489

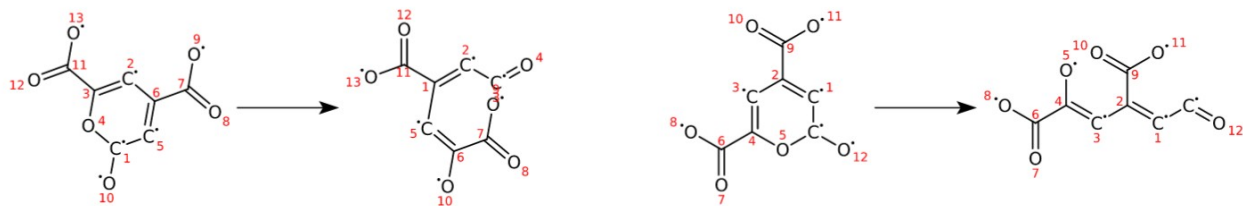

R05069

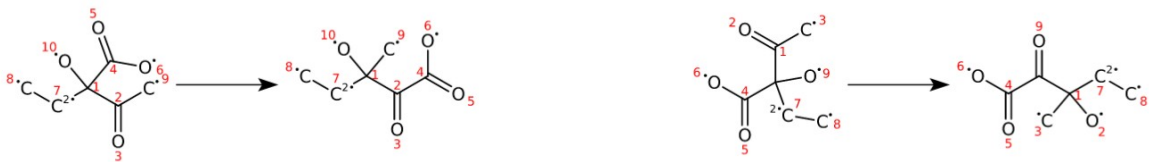

R05098

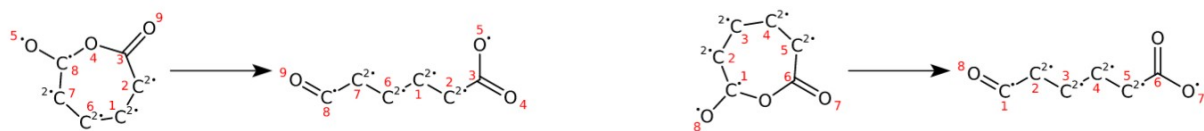

R05774

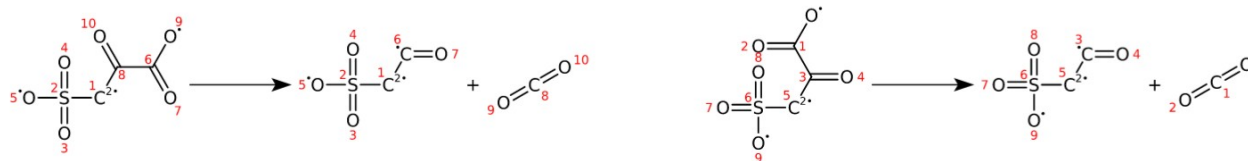

R07420

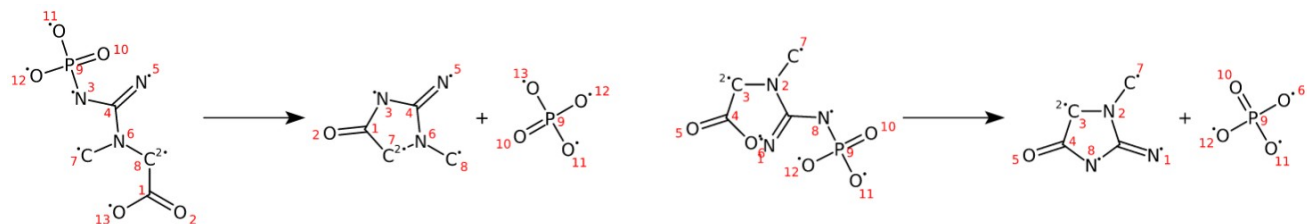

R08199

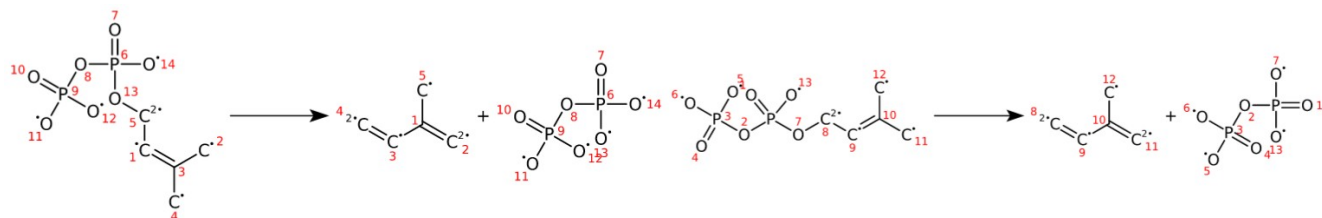

R08337

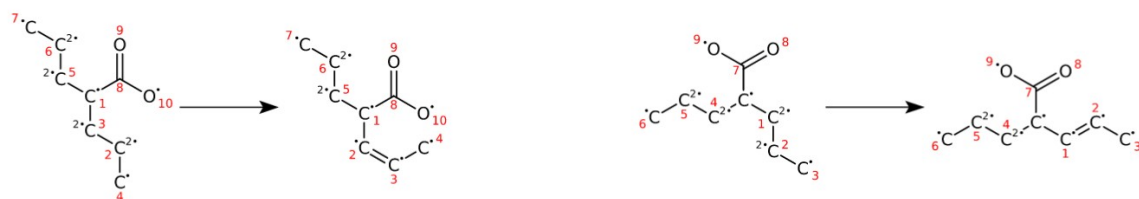

R08373

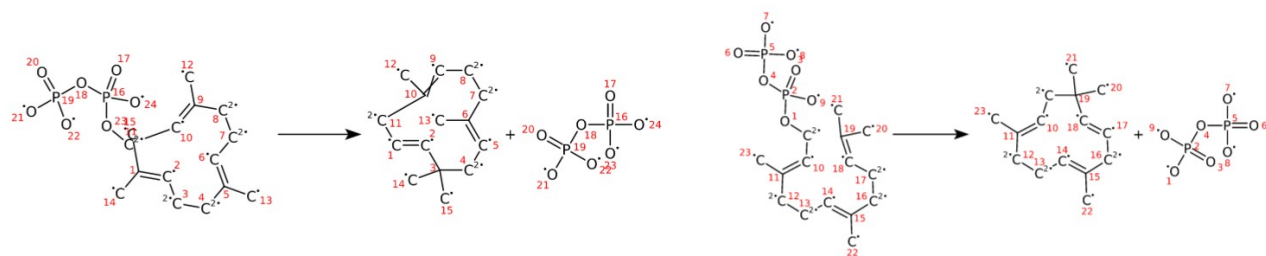

R11749

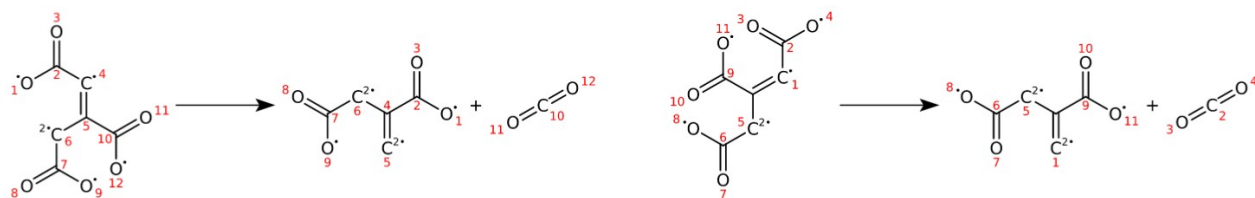

Supplement: Supplementary file 6 — (pdf 731 KB) [file 13015_2025_294_MOESM6_ESM.pdf]
