# Supplementary material for: Computing double-pushout graph transformation rules and atom-to-atom maps from KEGG RCLASS data
Source: Algorithms Mol Biol. 2026 Jan 29;21:3. doi: 10.1186/s13015-025-00294-6 (PMC12949509; doi:10.1186/s13015-025-00294-6)

N1a

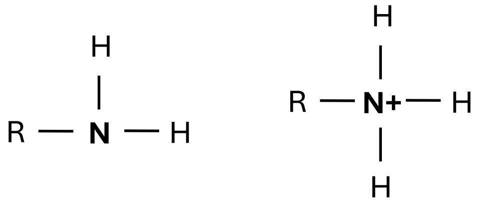

N1c

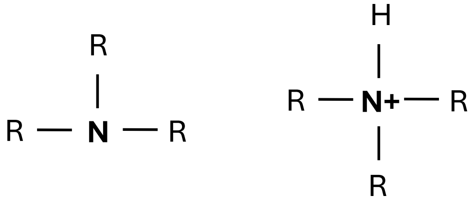

N1y

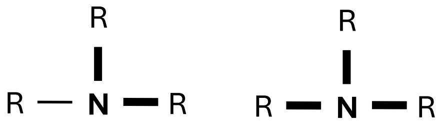

N2y

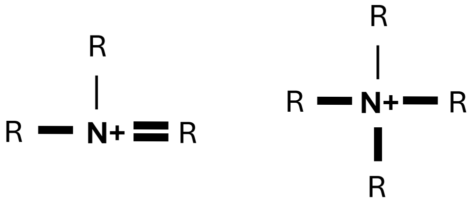

N2a

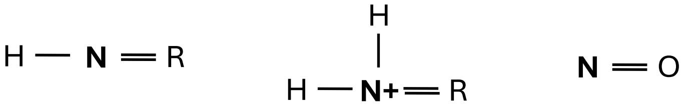

N3a

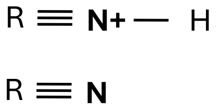

N2b

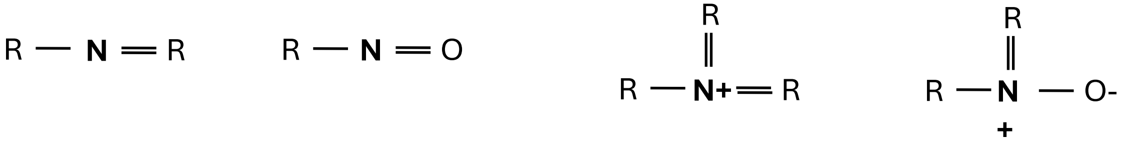

N1b

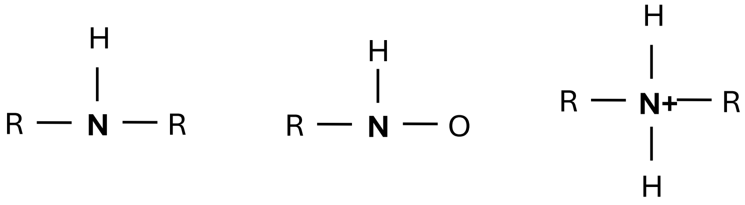

N4y

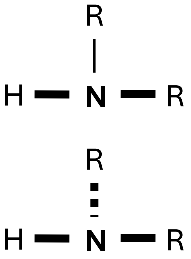

S4a

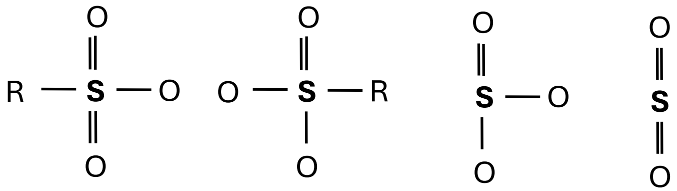

S3a

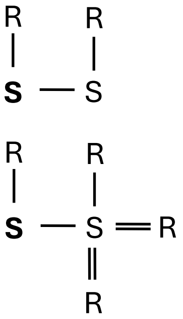

Legend

single bond

ring bond

dopple bond

triple bond

aromatic bond

S2x

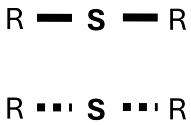

Supplement: Supplementary file 4 — (pdf 62 KB) [file 13015_2025_294_MOESM4_ESM.pdf]
